# Projecting Global Land-Use Change and Its Effect on Ecosystem Service Provision and Biodiversity with Simple Models

**DOI:** 10.1371/journal.pone.0014327

**Published:** 2010-12-15

**Authors:** Erik Nelson, Heather Sander, Peter Hawthorne, Marc Conte, Driss Ennaanay, Stacie Wolny, Steven Manson, Stephen Polasky

**Affiliations:** 1 The Natural Capital Project, Woods Institute for the Environment, Stanford University, Stanford, California, United States of America; 2 Conservation Biology Graduate Program, University of Minnesota, St. Paul, Minnesota, United States of America; 3 Department of Ecology, Evolution, and Behavior, University of Minnesota, St. Paul, Minnesota, United States of America; 4 Department of Geography, University of Minnesota, Minneapolis, Minnesota, United States of America; 5 Department of Applied Economics, University of Minnesota, St. Paul, Minnesota, United States of America; University of California, United States of America

## Abstract

**Background:**

As the global human population grows and its consumption patterns change, additional land will be needed for living space and agricultural production. A critical question facing global society is how to meet growing human demands for living space, food, fuel, and other materials while sustaining ecosystem services and biodiversity [1].

**Methodology/Principal Findings:**

We spatially allocate two scenarios of 2000 to 2015 global areal change in urban land and cropland at the grid cell-level and measure the impact of this change on the provision of ecosystem services and biodiversity. The models and techniques used to spatially allocate land-use/land-cover (LULC) change and evaluate its impact on ecosystems are relatively simple and transparent [2]. The difference in the magnitude and pattern of cropland expansion across the two scenarios engenders different tradeoffs among crop production, provision of species habitat, and other important ecosystem services such as biomass carbon storage. For example, in one scenario, 5.2 grams of carbon stored in biomass is released for every additional calorie of crop produced across the globe; under the other scenario this tradeoff rate is 13.7. By comparing scenarios and their impacts we can begin to identify the global pattern of cropland and irrigation development that is significant enough to meet future food needs but has less of an impact on ecosystem service and habitat provision.

**Conclusions/Significance:**

Urban area and croplands will expand in the future to meet human needs for living space, livelihoods, and food. In order to jointly provide desired levels of urban land, food production, and ecosystem service and species habitat provision the global society will have to become much more strategic in its allocation of intensively managed land uses. Here we illustrate a method for quickly and transparently evaluating the performance of potential global futures.

## Introduction

The earth's capacity to provide enough living space, food, and clean water to meet human needs as well as the habitat needs of other species is being severely tested [3]–[5]. A growing global human population and the associated increase in demand for living space, food, water, fuel, and other materials and services makes ecosystem service and biodiversity sustenance a difficult challenge. Having a clear understanding of how ecosystem service and habitat provision might change over time due to global urban and cropland development is a prerequisite for charting a global future that can meet these interconnected challenges [1]. In this paper, we develop methods for allocating expected areal changes in global land use/land cover (LULC) and for analyzing the likely consequences of these changes on the provision of several ecosystem services and species habitat.

Our approach provides a relatively simple and transparent method for creating spatially-explicit projections of global LULC change at the grid cell-level. Our spatial allocation of expected urban and cropland areal development is guided by rules that incorporate basic demographic, economic development, and biophysical principles. This method allows for the relatively quick creation of spatially-explicit projections of business-as-usual futures or alternative futures that might emerge if decision-making on urban and cropland development across the world changes, either due to shifts in consumption preferences or land-use policies.

We couple global LULC conversion scenarios with models that predict the consequences of these changes on the provision of crop, water availability, carbon storage in biomass (a climate regulation service), and habitat for species. Changes in ecosystem service provision are modeled using the Integrated Valuation of Ecosystem Services and Tradeoffs (InVEST) software system. InVEST is a suite of geographic information science models and algorithms that converts changes in LULC patterns into changes in terrestrial carbon storage, water availability, crop production, habitat for species, and other ecosystem service outputs (not all services modeled by InVEST are included in this illustration). Combining maps of alternative LULC futures with InVEST, we can estimate the range of potential changes in ecosystem service provision and tradeoffs among various services at different geographical and socioeconomic scales. These predictions can help frame the discussion of preferred global change outcomes and policy mechanisms needed to obtain them.

To illustrate our approach, we create two plausible scenarios of spatially-explicit LULC change for the period 2000 to 2015. To create a scenario we estimate global areal change in urban land and cropland from 2000 to 2015 and then spatially allocate the change at the grid cell-level. Cropland areal growth across the globe under the *country* scenario is given by extrapolating country-level 1985 to 2000 cropland growth trends to the 2000 to 2015 period [6]. Cropland areal growth across the globe under the *regional* scenario is based on estimates of regional growth in cropland area as given by the OECD-FAO's Agricultural Outlook trade model [7] where a region can be comprised of one country (e.g., the U.S.) or many countries (e.g., sub-Saharan Africa). Both scenarios assume the same level and pattern of urbanization [8]. Therefore, differences across the two scenarios are completely explained by divergent cropland development patterns. In this illustration, the area of grid cells classified as urban increases 23.6% across the globe between 2000 and 2015, a gain of 0.76 million sq. km^2^. The area of grid cells classified as cropland increases by 1.48 million sq. km^2^ (a 5.8% increase compared to 2000) under the *country* scenario and by 1.88 million sq. km^2^ (a 7.4% increase compared to 2000) under the *regional* scenario. The *country* scenario is distinguished by significant cropland expansion in China and Indonesia while the *regional* scenario is highlighted by significant cropland expansion in Brazil with net cropland abandonment in China.

We translate LULC changes under each scenario into changes in crop production, water availability, carbon storage in biomass, and species habitat using InVEST. The expansion of urban and cropland area leads to global declines in species habitat and biomass carbon storage. However, as measured by impact on global ecosystem services and habitat loss, the *country* scenario is superior to the *regional* scenario. First, the *country* scenario generates a greater increase in the caloric value of crop production. Second, under the *country* scenario the gain in caloric production is done more efficiently as measured by the tradeoffs between biomass carbon storage and species habitat provision. Specifically, under the *country* scenario 5.2 grams of biomass carbon is released due to LULC conversion for every additional calorie of crop produced. Under the *regional* scenario this tradeoff rate is 13.7. Further, under the *country* scenario 0.0016 square meters of species habitat is lost for every additional calorie of crop produced. Under the *regional* scenario this tradeoff rate is 0.0021.

The superiority of the *country* scenario is due primarily to two global development patterns. First, under the *country* scenario, large sources of biomass carbon storage are not converted to cropland when compared to the *regional* scenario. For example, significant cropland development in Brazil under the *regional* scenario, an important source of global biomass carbon stock, largely explains that scenario's relatively poor performance on the crop – carbon emissions tradeoff ratio. Second, under the *country* scenario there is greater cropland expansion in areas with greater agricultural technological and irrigation capacities, both important factors in sustaining continued increases in crop production efficiencies. For example, under the *country* scenario 62.3% of the net gain in cropland grid cell area is projected to be irrigated to some degree whereas under the *regional* scenario only 33.1% of net gain in cropland grid cell area is projected to be irrigated to some degree. These results highlight the general principle that the likelihood of meeting the joint challenge of sufficiently increasing food production while maintaining ecosystem service and species habitat provision will increase if we allocate cropland to areas of high or increasing agricultural productivity that do not also provide high levels of ecosystem services or important habitat. Developed countries currently have the highest agricultural productivity capacities and contain some of the least important sources of several ecosystem services and species habitat due to past development [9]. Therefore, if higher agricultural productivity capacities cannot effectively be transferred to the developing world then best hope for a sustainable future may be a reverse in the recent trend of minimal to no cropland growth in the developed world [6], [7].

Besides describing potential futures and their ramifications on ecosystem service and habitat provision, projections of LULC change can be used to inform policy [10]. We illustrate how our approach can be used to guide a policy that provides incentives to maintain carbon stocks in forest at risk for development (reducing carbon emissions from deforestation and forest degradation or REDD program [11]). Assuming that the scenarios described here are two examples of business-as-usual global development projections and using a plausible REDD policy framework, we estimate 0.1 billion metric tons of avoided carbon emission credits would have been generated across the globe from 2000 to 2015 under modest offset prices if the *country* scenario had been chosen as the baseline and 13 billion metric tons of avoided carbon emission credits would have been generated across the globe if the *regional* scenario formed the baseline. The stark differences in credit creation across the two scenarios illustrates how contentious the selection of a business-as-usual emission trajectory for any actual deforestation avoidance program may be [12], [13].

### Literature review: modeling global or regional LULC change at the grid cell-level

Spatially-explicit regional and global LULC change modeling has been the focus of several prominent research efforts. For example, the Millennium Ecosystem Assessment (MA) research team has developed four plausible projections of the earth's future. Each projection or scenario is defined by regional population, economic, and technological growth estimates as well as projections for food and energy demands to the year 2100 [14]–[16]. Using a set of climate, agricultural [17], water supply and use [18], [19], and LULC change models [20], [21], the MA team translated these expected regional change and demand trajectories into global grid cell-level LULC maps for the years 2050 and 2100 [22].

Instead of modeling change with such general equilibrium models, extent and pattern of LULC change can be generated by simulating the decision making of actors on the landscape. In agent-based modeling “agents” (e.g., households, firms, government agencies, etc.) make LULC decisions over time such that their preferences are maximized given land-use policy constraints and their neighbors' decisions [23], [24]. Alternatively, instead of simulating the behavior of agents through time, previous LULC change behavior on the landscape can be extrapolated into the future using one of several statistical techniques [25], [26], including cellular automata [27]–[29]. In agent-based modeling the challenge is getting the rules that guide agent behavior correct. The challenge with the statistical approach is 1) isolating and controlling for policy, biophysical, or economic conditions that shaped past decisions but will not exist in the future and 2) appropriately controlling for conditions that could affect future LULC decision making but were not present on the landscape in the past [30].

In other global and regional change research, the spatial allocation of LULC change is guided by a set of rules based on fundamental socioeconomic and biophysical principles. For example, McDonald et al. spatially allocate a United Nation's projection of country-level urban population growth across a grid using the notion that cities grow concentrically [31]. The California Urban Futures Model [32] allocates expected regional residential development such that undeveloped parcels modeled to have the highest profitability in residential land use are converted first. Alternatively, focus groups of appropriate experts and decision-makers have been used to codify the socio-economic, policy, and biophysical forces that drive LULC change across a region [33]–[35]. The model UPlan opens such rule-making ability to anyone [36]. In this GIS model users specify future population levels, demographic characteristics, and land-use density parameters. Given these inputs, area needed for each land-use is determined and then is spatially allocated according to user-defined or default land-use suitability maps. All of these approaches that use rules to spatially allocate LULC change tend to be simpler and more transparent than the general equilibrium scenario analysis as exemplified by the MA, agent-based modeling, or statistical analyses. However, these approaches do not verify that the resulting spatial patterns of LULC change are compatible with projected global or regional demands for food, energy, and other services or that the projected spatial patterns of change are consistent with past behavior.

### Literature review: estimating the impact of grid cell-level LULC change at global scales on environment, ecosystem services, and human well-being

Many analyses that estimate the environmental or human welfare impact of expected global LULC change work with maps where change is summarized at the country- or regional-level. Such analyses have been used to predict changes in global agriculture production, disease risk, energy use, species persistence, and water availability [3], [37]–[40]. However, such broad assessments ignore the heterogeneity in land uses and biophysical and economic conditions within regions.

To correct for such biases researchers are increasingly using grid cell-level assessments of global LULC change to project changes in the environment, ecosystem services, and human well-being. Such an approach is capable of capturing local-scale heterogeneity that is often important for determining the supply, demand, and value of ecosystem services. The MA project is a prominent example of this. The MA used already-published economic, biophysical, and ecosystem service models [41] to estimate the impact of their four 2100 grid cell-level LULC maps on the environment, ecosystem service production, biodiversity, and human well-being [22], [42]. The LULC change model used by the MA has been used in conjunction with other biophysical and climate models to generate global maps of predicted net primary productivity and climate modulation [43], land-use carbon emissions and other carbon cycle dynamics [44]–[47], trends in biodiversity [48], [49], food production [50]–[52], inorganic nitrogen export to coastal waters [53], and of various environmental conditions [54]. McDonald et al. estimate the impact of their future global urban area map on species persistence and protected areas in each terrestrial ecoregion on the globe [31]. Other ecosystem service models that can be used with global grid cell-level LULC maps are summarized in [55].

In this paper we spatially allocate already published country- or regional-level estimates of urban and cropland area change to the grid cell-level using a rules-based approach. The inclusion of cropland areal change and the use of land-use suitability matrices to guide LULC change extend this work beyond McDonald et al. 's work. Further, unlike, McDonald et al., we consider more than biodiversity impacts of LULC change around the globe. We produce some of the same output as the MA and other global ecosystem service analyses but without using some of the more complicated demographic, economic, and technological growth and biophysical models. In fact, the models we use from InVEST to model the impact of global change are open-source, freely available, and readily accessible (http://invest.ecoinformatics.org/).The simpler and more transparent approach lowers the barriers to participation in scenario building and ecosystem service provision modeling and allows for the quick and transparent assessment of future scenarios of change. We hope that a demonstration of our transparent and flexible method for modeling the potential ramifications of global change leads to wider use of our or similar modeling approaches by policy-makers throughout the world [56], [57].

### Our method for estimating the impact of grid cell-level LULC change at global scales on environment, ecosystem services, and human well-being

We spatially allocate projected country- or regional-level 2000 to 2015 net change in urban and cropland area to the grid cell-level (5 km resolution at the equator). Country-level urbanization projections for 2015 are based on urban population expansion estimates from the United Nations [8]. We spatially allocate two different projections of cropland areal change. The first projection of change is generated by extrapolating the rate of country-level cropland area change from 1985 to 2000 to the 2000 to 2015 time period (the *country* scenario). In the other cropland change scenario (the *regional* scenario), we use the OECD-FAO's Agricultural Outlook trade model [7] to estimate 2015 cropland area targets at the regional-level. The spatial extent and pattern of cropland change varies across the two scenarios because of differences in expectations for areal change and the geographic unit of analysis. For example, in the *regional* scenario cropland growth is greater in developing countries than it is under the *country* scenario.

Spatial allocation of expected urban and cropland change is done using a cellular modeling technique [58], [59]. Under this technique, urban expansion between 2000 and 2015 tends to occur in cells that are near urban land as of 2000 and that have higher urban suitability scores. A cell's urban suitability score 1) increases in its projected 2015 population density [60] and 2) decreases in its slope [61]. Urban expansion into protected areas is not allowed [62].

Then we use the cellular modeling technique and a cropland suitability map to spatially allocate a scenario's expected country-level or regional-level net growth in cropland area (or abandonment if appropriate) to the grid cell-level. Cropland expansion only occurs on land that is arable, is not in protected areas, and has not been allocated to urban expansion (i.e., we assume that urban land expansion will outbid cropland expansion on land highly suitable for both [4]). The cropland suitability map has higher scores in grid cells where 1) cereal yield potential under intensive management, including irrigation if applicable, is higher [63] and 2) slopes are gentler [61]. Potential cereal yield tends to be higher in areas with fecund soil, that has sufficient water (either due to rainfall, irrigation, or both), and have temperate climatic conditions. In the end we generate two gridded maps of LULC as of 2015 where urban grid cell extent and pattern is the same across both maps but cropland grid cell extent and pattern differ.

Even though we believe our suitability maps have captured the basic principles that drive urban and cropland change (in the urban case, we are adopting the basic principles of change assumed by [60]), we acknowledge that our suitability maps ignore many dynamics that guide LULC change. For example, infrastructure development plays a key role in both urban and cropland conversion [64]; our suitability maps do not explicitly capture existing and potential infrastructure development, investments that could increase suitability (e.g., reclaiming land through drainage or other means), and other forces of change. In the Discussion section below we note how our cropland suitability layer could be improved to capture the various forces of change that we have not included. For now we view our methodology for allocating estimated global LULC change at the grid cell-level as a first generation model that can be improved over time.

We estimate how the projected global change in LULC extent and pattern from 2000 to 2015 will affect the global provision of crops, water availability, carbon storage in biomass, and habitat for species with the appropriate InVEST models. To calculate change in global cropland production we first need to calculate change in annual harvested hectares in each country between 2000 and 2015 (we complete this and all subsequent methodological tasks for both scenarios). This calculation is a function of fallow land practices and the intensity at which the land is cropped in a country in 2000 and 2015. Next, we convert a country's pattern of change in harvested area into a change in the country's crop production (measured in both mass and caloric terms) using the InVEST agriculture model. In addition to change in harvest area, country-level change in crop production is a function of the relative change in the potential productivity of land used in the country for crop production, expected technological and infrastructure growth in the country's agricultural sector, and the mix of crops grown in the country [7], [65]. The overall productivity of the land used for cropland in a country as of 2015 will change compared to 2000's overall productivity if cropland expansion occurs on land with different yield potentials than that of cropland as of 2000. In general, if 1) the country's spatial allocation of cropland in 2015 is located on land with greater yield potential than its 2000 allocation (which can be aided by the expansion of irrigation) and/or 2) the country is expected to benefit to from yield growth in crops that dominate its 2015 crop mix, then growth in its crop production will outpace its growth in harvested area. We summarize change in crop production by country under both scenarios.

Predictions of cropland and crop production change, both in magnitude and spatial pattern, are uncertain for many reasons and we highlight a few of them here (see the Materials and Methods section for a complete discussion on sources of uncertainty). Crop yield potential is limited by water availability. Our cropland suitability layer and the base yields (observed year 2000 yields) used in the crop production model are based on water availability trends of the late 20^th^ century (and irrigation patterns when it comes to base yields). Climate change and LULC change may result in shifts in water yield (precipitation less evapotranspiration), thereby making the cropland suitability layer and base yield estimates imperfect predictors of 2015 patterns in cropland productivity and base yields. One way we can begin to assess how crop production may deviate from modeled expectations due to climate change and LULC-driven changes in evapotranspiration is to predict average annual water yield (average annual rainfall less annual evapotranspiration in mm km^−2^) on each cropland grid cell in 2015. Specifically, we model average annual water yield in 2000 and 2015 on each cropland grid cell in 2015 with use HadCM3 climate model [66] and the InVEST water yield model [67]–[72] (we model 2000 yield instead of using actual data to keep comparisons between 2000 and 2015 consistent). If average annual water yield on a grid cell decreases over time then the cell's expected productivity as given by late 20^th^ century climate patterns may be too high, especially if the cell primarily contains rainfed cropland. Further, a decrease in a cell's yield reduces the runoff that can be used for irrigation by other cropland grid cells downstream. With this map of annual water yield, we can identify the portions of the world where estimated crop productivity estimates, especially rainfed productivity, may be particularly vulnerable to climate change as of 2015 and beyond.

Cereal yield potential on a grid cell is partly explained by irrigation; in general, potential yield in a cell is higher if we assume a significant portion of cropland in the cell is irrigated instead of rainfed. Therefore, in this illustration, areas more likely to be irrigated in the future are more likely to be selected for cropland expansion (recall that the suitability score of a grid cell is largely determined by its relative potential for cereal production). We assign an arable cell in a country its irrigated yield potential instead of its rainfed potential on the cropland suitability layer if it has a yield potential profile that closely matches the yield potential profile of cells with significant irrigation in the country as of 2000 [73]. This modeling process creates a yield potential map where most cells that were significantly irrigated in 2000 are assigned their irrigated yield potential (we model the 2000 irrigation patterns instead of using the observed pattern to keep comparisons between 2000 and 2015 consistent). In addition, some arable cells in each country not in cropland as of 2000 but that closely resemble their country's irrigated cells as of 2000 are given their irrigated rather than rainfed yield potential. How similar these arable cells have to be to those that were significantly irrigated in 2000 to receive their irrigated yield potential is determined for each country by the modeler (SI Text 1). The less strict the resemblance required, the greater the number of arable cells in a country that are assigned their irrigated yield potential. In the end, if irrigation infrastructure and technology is not implemented in the pattern assumed by our cropland suitability layer then the spatial allocation of cropland expansion and modeled relative change in cropland productivity will be inaccurate, especially in those countries expected to rely heavily on irrigation to fuel their crop production growth (see Table S1 for country-by-country estimates of growth in cropland grid cell area that will benefit to some degree by irrigation under both scenarios).

While we assume that cropland output in a cell is solely a function of its productive potential and access to water, there are many other factors that affect the productivity of cropped areas, including infrastructure designed to support agriculture. For example, Vera-Diaz et al. show that soybean yields are higher, all else equal, if the farmer has access to markets via roads [74]. They surmise that farmers invest more of their time and capital in cropping operations if they can easily market their product. Presumably such infrastructure has been established in areas where cropland has existed for sometime. Newly established croplands, however, may not have the infrastructure necessary to support maximum production effort. Therefore, the InVEST agriculture production model includes a term that adjusts production on newer croplands according to infrastructure capacity and other factors (e.g., experimentation with fertilization rates to find the most cost-effective application) that might prevent maximum production capacity immediately. However, we do not use this term it in this illustrative example due to a lack of global data on the relationship between agriculture infrastructure and yields.

It is also possible that arable area around the globe could be expanded in the future. For example, draining low-lying areas (e.g., polders in the Netherlands) can increase the base of arable land. Conversion of existing agricultural to urban land and increased demand for food from a growing population will increase the incentive to make such investments. However, we lacked systematic data on which to base an assessment of an expansion of arable land base through investment and so did not include this dynamic in our analysis.

We use the carbon sequestration InVEST model to measure the change in biomass carbon storage (carbon sequestration) in each grid cell due to LULC change. First, we find average biomass carbon storage levels for each LULC type for each country using mapped Intergovernmental Panel on Climate Change storage data and the 2000 LULC map. Second, we apply these average storage values to the maps of LULC in 2000 and 2015. The difference in each grid cell's storage value between 2000 and 2015 gives a gridded map of change in storage. (Because it can take decades for a LULC type to reach its average biomass carbon storage level we do not technically measure actual biomass carbon sequestration over the 2000 to 2015 period; instead we measure the eventual change in biomass carbon storage if the 2015 global LULC map were maintained indefinitely.) Our application of the InVEST sequestration model does not account for biomass carbon flux in grid cells that do not experience a LULC change, it does not account for carbon flux due to land management, and we do not attempt to adjust storage capacities and sequestration rates due to expected climate change. We summarize biomass carbon sequestration results by country under both scenarios.

We also measure the conversion of undeveloped land by ecoregion [75], [76]. In our analysis we make the simplifying assumption that undeveloped land – all LULC types other than urban and cropland – is more likely to provide habitat for species than urban and cropland area. Therefore, changes in undeveloped land area are correlated with change in species habitat. (Our inability to translate mapped land covers into habitat types and a lack of a comprehensive, global dataset on species-land cover suitabilities makes it impossible for us to model changes in global habitat; see the Discussion section for more details.) To identify which scenario is more detrimental to species persistence we summarize loss of undeveloped land area at the ecoregion level and then cross-walk these losses against measures of ecoregion habitat availability, habitat connectivity, and numbers of endangered and threatened species.

We combine projections in LULC change and carbon storage maps to make a contribution to the recent policy discussions on REDD. Under REDD or some similar avoided emissions policy, countries that reduce deforestation or forest degradation below some business-as-usual rate would generate avoided carbon emission credits that could be sold to entities looking to reduce their carbon emission liabilities. LULC change scenarios that do not assume a REDD policy, like those presented here, could be used by policy makers to predict business-as-usual country-level deforestation and associated emissions rates. To simulate a representative avoided deforestation global policy illustrate this process, we estimate the area of forest in each country that is cleared under a scenario but, given a sufficient avoided deforestation payment, would have generated more in economic returns by accepting the avoided deforestation payment than converting to the projected land use (subject to a country cap on avoided emission credits that is a function of historic deforestation rates [77]). Because we assume the economic value of urban expansion will always be greater than the value of any avoided emission credit, avoided deforestation and credit generation only occurs in grid cells where cropland is predicted to emerge from forest between 2000 and 2015 but the value of the credit is greater than the expected value of the new cropland. We summarize avoided emission results by country under both scenarios. (Obviously, if the avoided deforestation policy was implemented as given and occurred as modeled then changes in crop production, water yield, biomass carbon emissions, and loss in undeveloped land from 2000 to 2015 would be different than given here.)

We order country-level results according to a measure of human development, the 2006 Human Development Index (HDI; [78]). We do this to identify the spatial correlations between patterns of global change and current human well-being [5], [79]. HDI, which ranges from 0 to 1 where higher scores indicate greater overall human well-being in a country, is a composite measure of a country's life expectancy, educational attainment, and per capita GDP. HDI scores are often ranked in descending order where the country with the highest HDI has a ranking of 1.

## Results

### Urbanization and cropland grid cell area change

**Figure 1:**
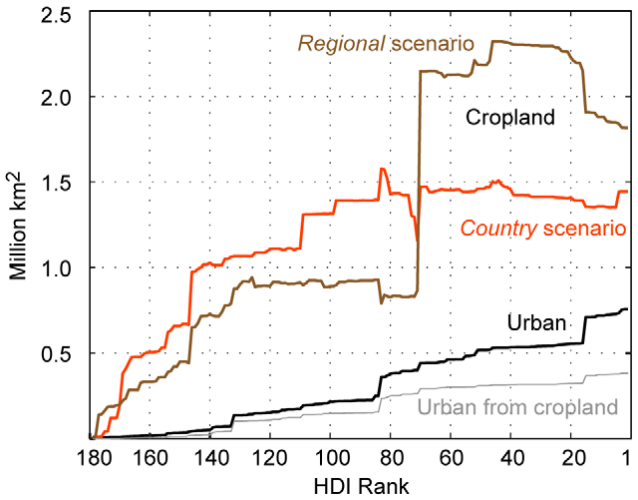
Projected net change in global urban and cropland grid cell area from 2000 to 2015. Country-level contribution to projected global net change in urban and cropland grid cell area is sorted by 2006 Human Development Index (HDI) rank. There is one projection of urban grid cell area change and two projections of cropland grid cell area change (the *country* and *regional* scenarios). The graph also indicates urban grid cell area that was established between 2000 and 2015 on cropland grid cells. The portions of the cropland grid cell area curves that decline indicate countries expected to experience a net decline in cropland grid cell area. This graphic does not include the 0.007 million km^2^ gain in urban grid cell area in unclassified HDI countries. This graphic does not include the 0.03 and 0.07 million km^2^ net gain in cropland grid cell area in unclassified HDI countries under the *country* and *regional* scenarios, respectively.

We plot cumulative country-level net change in urban and cropland grid cell area between 2000 and 2015 from lowest to highest HDI score (Figure 1). The area of grid cells classified as urban expands by 0.76 million km^2^, roughly the size of Turkey (expansion in urban grid cell area does not equal expansion in urban area as many grid cells that are primarily urban will include some other land covers as well). Depending on the scenario, global cropland grid cell area is expected to expand by 1.48 to 1.88 million km^2^ over this time period, roughly the size of Iran and Libya, respectively (again, expansion in cropland grid cell area does not equal expansion in croplands as many grid cells that are primarily cropland will include some other land covers as well). This graph understates the amount of new cropland grid cell area that will emerge across the globe as additional cropland will be needed to compensate for the cropland that existed as of 2000 but converts to urban area as of 2015. Most of the growth in cropland is located in the least-developed countries (Brazil and Libya under the *regional* scenario are the exceptions).

### Crop production services

**Figure 2:**
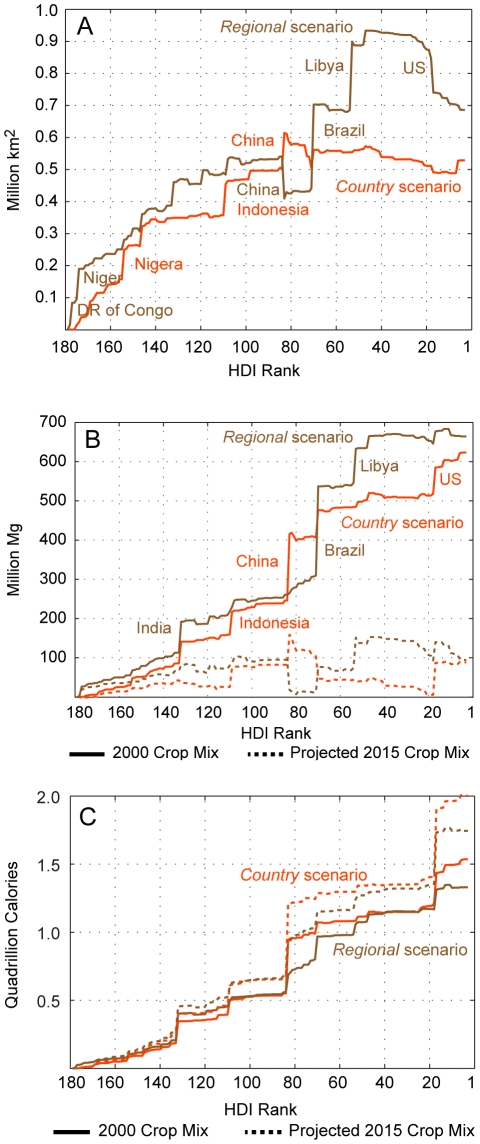
Projected net change in global harvested area and crop production from 2000 to 2015. Country-level contribution to projected global net change in harvested area (panel A) and crop production measured in mass (panel B) and calories (panel C) is sorted by 2006 HDI rank for both scenarios. Countries projected to experience significant changes are noted. For panels B and C scenario results are given once assuming each country's 2000 crop mix remains as of 2015 (“The 2000 Crop Mix”) and once assuming 2015 crop mixes mimic forecasted trends in crop mix (“Projected 2015 Crop Mix”). By crop mix we mean the relative amount of harvested area devoted to each crop type (e.g., rice, wheat, oil crops, etc.) in a country. Panel C does not include a country's production of crops in the categories fiber crops, oil seeds, and other oil crops. The portions of the curves that decline indicate countries expected to experience a net decline in crop production on the given metric. This graphic does not include the net gain in harvested area and crop production in unclassified HDI countries.

Change in harvested area strongly mirrors change in cropland grid cell area; however, it is not a perfect predictor of change in harvested hectares due to differences across the globe in cropping intensity, fallow practices, and the matrix of land used for other purposes in grid cells designated as cropland (see the Methods and Materials section for information on how we converted change in cropland grid cell area to change in harvested hectares). Like the change in cropland grid cell area, net growth in harvested hectares in countries with low HDI scores is stronger under both scenarios (panel A of Figure 2). These results are consistent with expectations that developing countries will produce an increasing share of the world's crops in the future [7]. As with the graph of the net change in cropland grid cell area, this graph understates the amount of new harvested area that will emerge across the globe as additional harvested area will be needed to compensate for the harvested area that existed as of 2000 but is converted to urban area by 2015.

While growth in harvested area can be a significant driver of economic growth in an area [80], change in crop production most directly impacts human well-being. All else equal, a net increase in area devoted to crop production in a country will increase its output over time (whether measured in mass or calories). In addition, a country's production will get a boost over time if it grows crops that are expected to experience yield increases due to improvements in agricultural methods and technology. Production levels in a country can also be positively affected by certain patterns of cropland change; namely, if a country's 2015 pattern of cropped area covers grid cells that, on average, have greater yield potential than the cropped area in 2000 then, all else equal, production will increase. A change in a country's crop mix between 2000 and 2015 also affects production (by crop mix we mean the relative amount of harvested hectares devoted to each crop type, e.g., rice, wheat, oil crops, etc., in a country). If a country switches to more dense crops, either in terms of mass or calories, then, all else equal, its production (as measured by mass or calories) will increase. If a country switches towards crops whose yields are improving due to technology managerial improvements then, all else equal, its production (as measured by mass or calories) will increase.

In panel B of Figure 2 we present modeled change in the mass of crop production by country, once under the assumption that the 2015 crop mix in a country mimics its 2000 mix (labeled “2000 Crop Mix”) and another time under the assumption that country-level crop mixes as of 2015 are more in line with forecasts from [7] (labeled “Projected 2015 Crop Mix”). In Table 1 we present more detailed information on the modeled changes in the mass of production at the country-level under the assumption that the 2015 crop mix in a country mimics its 2000 mix. In this case the increase in the mass of crop production in countries with low HDI (0 to 0.5) is primarily due to the increase in cropped area (and any improvements due to a potentially more productive pattern of cropland) while the increase in the mass of crop production in countries with medium and high HDI (0.5 to 0.8 and 0.8 to 1, respectively) is increasingly explained by improved yields, either due to technological improvement, increased irrigation capacity, the use of more productive land, or all three. Change in the mass of production in low and medium HDI countries is more efficient under the *regional* scenario (as measured by the ratio of change in net output to net change in harvested area). This result reflects the fact that the cellular modeling technique generally has more freedom to choose the most suitable cropland grid cells under the *regional* scenario than it does under the *country* scenario (i.e., the cellular model is allocating over a region rather than a country). However, because abandonment of cropland area in the highest HDI countries (nations with better access to technological improvements in the agricultural sector) is much smaller under the *country* scenario, the difference in crop production growth between the scenarios is small despite the *regional* scenario's additional 0.19 million harvested km^2^ (by 2015 the mass of crop production under the *country* scenario is 10.3% greater than 2000 modeled production whereas the gain is 11.2% under the *regional* scenario). In Table 2 we present information on the ratio of change in net output to net change in harvested area when we use 2015 country-level crop mixes that are more in line with forecasts from [7]. While the trends across HDI groups that we observed in Table 1 are similar, the global change in the mass of crop production under the *country* and *regional* scenarios are significantly lower, 1.5% and 1.7%, respectively. In other words, future crop mixes are forecasted to be much lighter from a mass perspective than the mixes found in 2000.

**Table 1:** Change in the mass of crop production between 2000 and 2015 assuming country-level crop mixes in 2015 mimic those observed in 2000.

		*Country* scenario				*Regional* scenario			
HDI Country Group	No. of countries	Net change in million harvested km^2^	Change in production (million Mg)	Change in prod. /change in harvested km^2^ (Mg/km^2^)	% of countries where new cropland is, on average, more productive than 2000 cropland	Net change in million harvested km^2^	Change in production (million Mg)	Change in prod. /change in harvested km^2^ (Mg/km^2^)	% of countries where new cropland is, on average, more productive than 2000 cropland
Low	26	0.25	49	194	0.16	0.28	68	243	0.38
Medium	78	0.33	359	1,102	0.33	0.15	228	1,520	0.68
High	73	−0.05	215	NA	0.39	0.26	368	1,443	0.59

Note: Countries that do not have a 2006 HDI are not included in this table.

**Table 2:** Change in the mass of crop production between 2000 and 2015 using 2015 projected country-level crop mixes in 2015.

	*Country* scenario		*Regional* scenario	
HDI Country Group	Change in production (million Mg)	Change in prod. /change in harvested km^2^ (Mg/km^2^)	Change in production (million Mg)	Change in prod. /change in harvested km^2^ (Mg/km^2^)
Low	23	91	40	141
Medium	96	294	−26	NA
High	−30	NA	81	316

Note: Countries that do not have a 2006 HDI are not included in this table.

While measuring crop production by mass is appropriate for economic analyses of production, measuring production in calories is a better indicator of the value that crop production provides to human well-being [81]. If we convert crop production from mass units to calories [82] global production under the projected 2015 crop mix [7] outperforms the 2000 mix in both scenarios (panel C of Figure 2 and Table 3; we exclude oil and fiber crops from the analysis). The superiority of the projected crop mix in caloric terms again suggests that lighter but more energy dense grains are expected to become more and more dominant in global food production. Further, the *country* scenario now outperforms the *regional* scenario on the global crop production metric. This suggests that the expansion of global harvested area under the *country* scenario tends to occur in countries expected to experience more significant yield growth in grains.

**Table 3:** Change in the caloric value of crop production between 2000 and 2015.

	*Country* scenario				*Regional* scenario			
	2000 Country-Level Crop Mixes		Projected 2015 Country-Level Crop Mixes		2000 Country-Level Crop Mixes		Projected 2015 Country-Level Crop Mixes	
HDI Country Group	Change in production (trillion calories)	Change in production/change in harvested km^2^ (million calories/km^2^)	Change in production (trillion calories)	Change in production/change in harvested km^2^ (million calories/km^2^)	Change in production (trillion calories)	Change in production/change in harvested km^2^ (million calories/km^2^)	Change in production (trillion calories)	Change in production/change in harvested km^2^ (million calories/km^2^)
Low	87	346	109	436	103	368	124	442
Medium	895	2,744	1,141	3,499	658	4,388	886	5,909
High	555	NA	752	NA	569	2,230	734	2,877

Note: Countries that do not have a 2006 HDI are not included in this table.

Whether the projected increase in calorie production is adequate to meet expanding human demand, both as food consumed directly and as feed for livestock, is uncertain. Global population is projected to increase by 19.1% from 2000 to 2015 [8] while caloric production in crops is expected to increase from 16.0% to 23.8% (the *regional* scenario with the 2000 crop mix and the *country* scenario with the projected 2015 crop mix; calorie growth does not include the growth in the production of meat, eggs, and milk from livestock supported by pasture and rangeland vegetation). However, even if global caloric output is sufficient to keep pace with population, pockets of malnourishment are likely to persist due to uneven distribution of crop production across regions [83].

### Annual water yield on rainfed cropland

**Figure 3:**
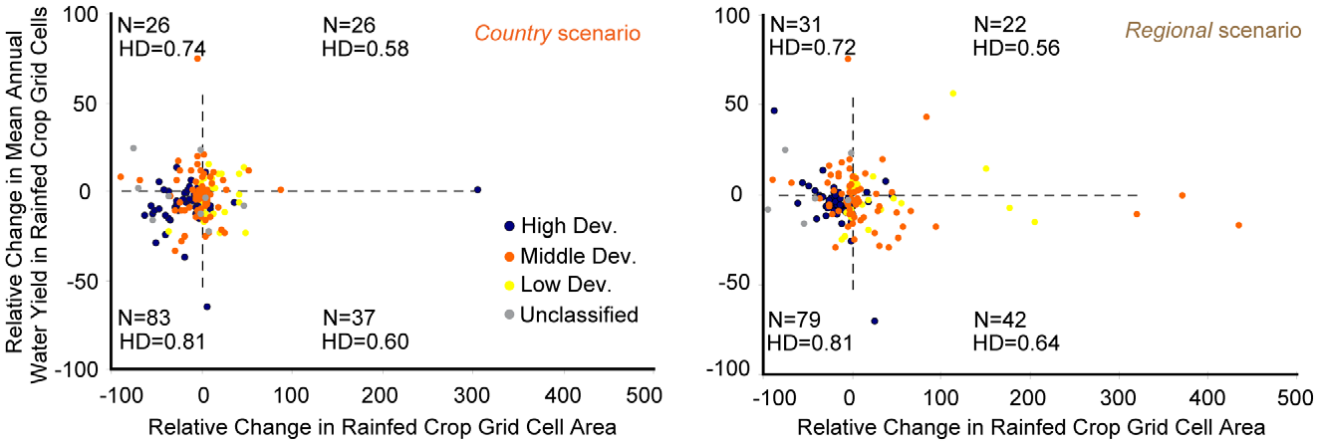
Projected relative change in rainfed cropland grid cell area versus relative change in average annual water yield on rainfed cropland grid cells. Panel A gives results for the *country* scenario. Panel B gives results for the *regional* scenario. Each point represents a country. A high development country (“High Dev.”) has a 2006 HDI greater than or equal to 0.8, a middle development country (“Middle Dev.”) has a 2006 HDI greater than or equal to 0.5 but less than 0.8, a low development country (“Low Dev.”) has a 2006 HDI less then 0.5, and a unclassified country has no HDI score. We indicate the number of countries (N) and the average HDI score (HD) of those countries in each quadrant in each graph.

Whether there will be sufficient water to support all modeled growth in crop production is a major concern. To begin to explore this issue we plot country-level changes in rainfed cropland grid cell area (cropland in cells assigned the potential rainfed yield on the cropland suitability layer) versus the projected change in average annual water yield on rainfed cropland grid cells (Figure 3). Production on rainfed cropland is limited by water produced directly on the cropland and cannot be maintained by irrigation. Therefore, our projections for crop production growth may be too high in countries that are projected to expand rainfed cropland area but experience, on average, a decline in annual water availability (the lower right quadrant of the graphs in Figure 3 indicates countries with such a tradeoff). There are five more countries in the lower right quadrant under the *regional* scenario (panel B) than in the *country* scenario (panel A). Further, projected rainfed cropland expansion is quite dramatic in several of these at-risk countries under the *regional* scenario (see Tables 4 and 5 for a list of all countries in the lower right quadrants of the graphs in Figure 3).

**Table 4:** Countries where rainfed cropland area increases but average annual water yield on rainfed cropland area decreases between 2000 and 2015 under the *country* scenario (the lower right quadrant of panel A in Figure 3).

Country	HDI	HDI Country Group	Percentage change in annual water yield on rainfed cropland grid cells	Percentage change in rainfed cropland grid cell area
Congo, DRC	0.361	Low	−5.1	0.5
Mozambique	0.366	Low	−6.3	41.8
Guinea-Bissau	0.383	Low	−11.7	25.6
Mali	0.391	Low	−6.7	2.8
Guinea	0.423	Low	−1.8	40.8
Rwanda	0.435	Low	−8.8	2.7
Zambia	0.453	Low	−17.5	3.5
Malawi	0.457	Low	−23.1	48.9
The Gambia	0.471	Low	−23.8	20.6
Angola	0.484	Low	−12.6	4.5
Uganda	0.493	Low	−12.3	14.0
Senegal	0.502	Medium	−23.3	8.1
Tanzania	0.503	Medium	−14.1	9.5
Kenya	0.532	Medium	−21.3	21.7
Mauritania	0.557	Medium	−23.4	26.1
Cambodia	0.575	Medium	−7.5	4.5
Myanmar	0.585	Medium	−1.0	3.0
Laos	0.608	Medium	−3.8	3.5
Bhutan	0.613	Medium	−5.9	4.4
Congo	0.619	Medium	−8.2	5.2
Botswana	0.664	Medium	−25.4	4.2
South Africa	0.67	Medium	−15.8	4.5
Nicaragua	0.699	Medium	−0.4	2.4
Bolivia	0.723	Medium	−1.5	9.3
West Bank	0.731	Medium	−8.7	19.1
Tunisia	0.762	Medium	−4.3	6.9
Georgia	0.763	Medium	−7.5	5.6
Jordan	0.769	Medium	−4.0	3.1
Peru	0.788	Medium	−5.1	5.4
Bosnia & Herzegovina	0.802	High	−10.9	0.4
Brazil	0.807	High	−8.4	8.9
Serbia & Montenegro	0.821	High	−10.2	10.4
Oman	0.839	High	−64.7	6.0
Latvia	0.863	High	−6.2	35.8
Iraq		Undefined	−3.9	3.6
San Marino		Undefined	−8.1	46.6
Zimbabwe		Undefined	−22.6	7.8

**Table 5:** Countries where rainfed cropland area increases but average annual water yield on rainfed cropland area decreases under the *regional* scenario (the lower right quadrant of panel B in Figure 3).

	HDI	HDI Category	Percentage change in annual water yield on rainfed cropland grid cells	Percentage change in rainfed cropland grid cell area
Congo, DRC	0.361	Low	−7.2	176.2
Mozambique	0.366	Low	−7.9	32.2
Niger	0.37	Low	−2.3	45.7
Guinea-Bissau	0.383	Low	−15.0	205.7
Mali	0.391	Low	−5.4	11.1
Guinea	0.423	Low	−5.0	54.7
Malawi	0.457	Low	−19.3	18.1
Angola	0.484	Low	−3.1	17.4
Senegal	0.502	Medium	−24.9	1.9
Tanzania	0.503	Medium	−15.9	0.9
Kenya	0.532	Medium	−28.3	30.8
Madagascar	0.533	Medium	−21.5	25.4
Mauritania	0.557	Medium	−28.9	41.8
Cambodia	0.575	Medium	−10.5	50.8
Myanmar	0.585	Medium	−1.8	5.4
Laos	0.608	Medium	−3.9	3.7
Bhutan	0.613	Medium	−5.9	4.4
Congo	0.619	Medium	−0.1	371.5
Namibia	0.634	Medium	−18.0	94.7
Botswana	0.664	Medium	−18.0	57.4
Uzbekistan	0.701	Medium	−6.1	31.3
Moldova	0.719	Medium	−12.8	1.7
Guyana	0.725	Medium	−21.4	1775.2
Gabon	0.729	Medium	−10.8	319.2
West Bank	0.731	Medium	−8.8	28.9
Paraguay	0.752	Medium	−13.0	13.7
Azerbaijan	0.758	Medium	−24.3	52.1
Tunisia	0.762	Medium	−4.3	6.9
Georgia	0.763	Medium	−9.4	66.5
Jordan	0.769	Medium	−4.0	3.1
Suriname	0.77	Medium	−17.3	435.0
Armenia	0.777	Medium	−10.9	0.9
Ukraine	0.786	Medium	−14.0	9.0
Bosnia & Herzegovina	0.802	High	−10.7	1.0
Kazakhstan	0.807	High	−5.8	0.4
Belarus	0.817	High	−11.5	1.4
Oman	0.839	High	−70.4	26.0
Libya	0.84	High	−87.8	1099.0
Uruguay	0.859	High	−4.2	17.6
Brunei	0.919	High	−0.8	45.1
Somalia		Undefined	−15.7	7.6
Zimbabwe		Undefined	−22.3	4.9

**Figure 4:**
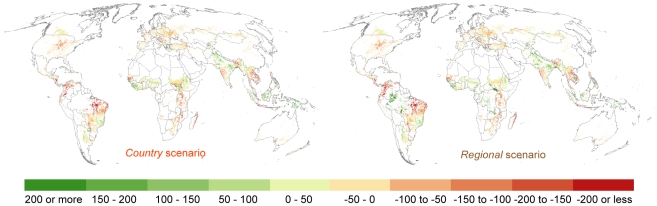
Projected change in average annual water yield from 2000 to 2015 on 2015 rainfed cropland grid cells around the globe (mm km^−2^). The map on the left gives results for the *country* scenario while the map on the right gives results for the *regional* scenario. White areas on a map either mean there is no change in water yield over time or the area is not identified as a rainfed cropland grid cell as of 2015.

**Figure 5:**
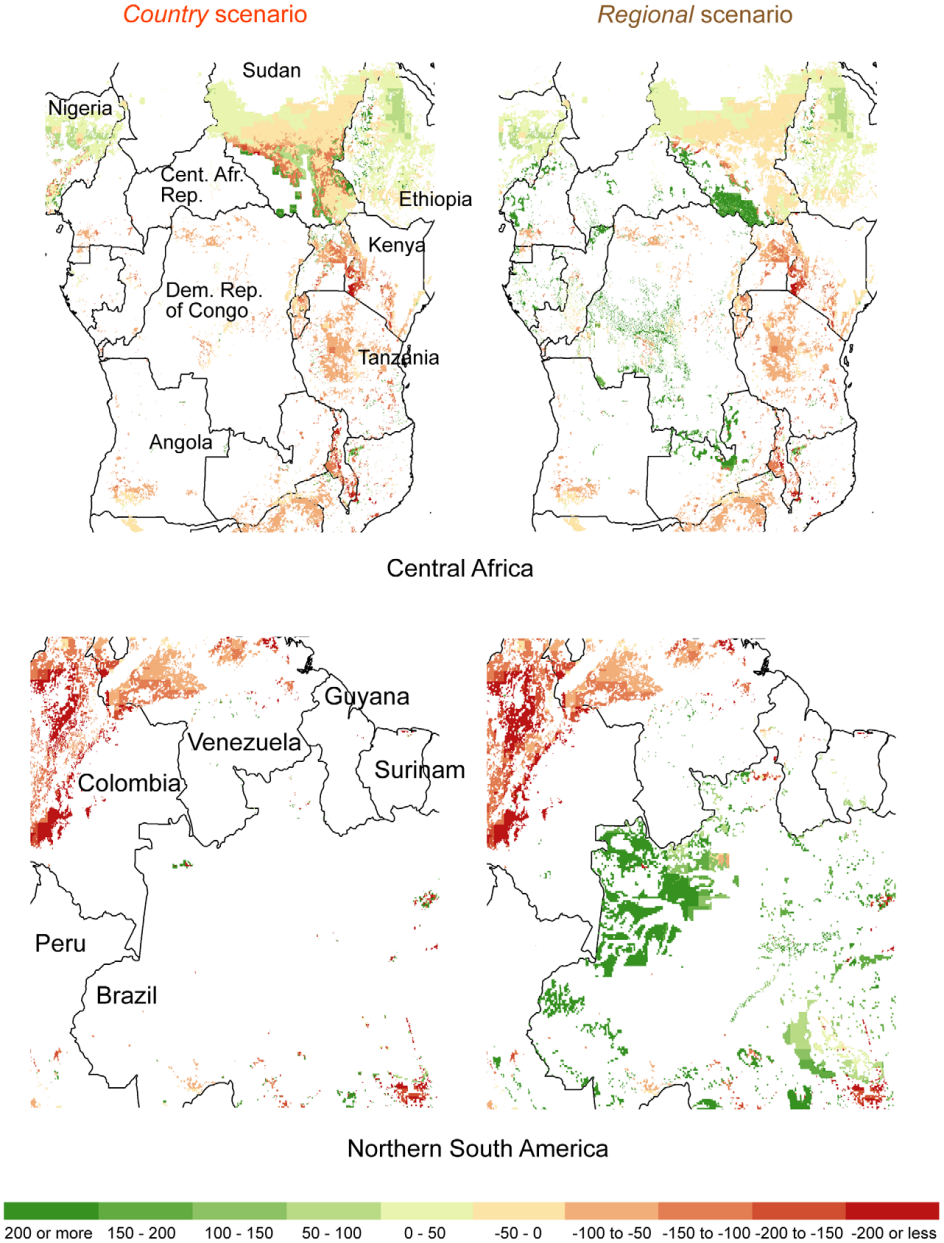
Projected change in average annual water yield from 2000 to 2015 on 2015 rainfed cropland grid cells for two regions (mm km^−2^). The maps on the left give results for the *country* scenario while the maps on the right give results for the *regional* scenario. White areas on a map either mean there is no change in water yield over time or the area is not identified as a rainfed cropland grid cell as of 2015.

Despite having more countries where, on average, water yield on rainfed cropland grid cells is decreasing while the area of rainfed cropland grid cells is increasing, from a global perspective the expansion in rainfed cropland under the *regional* scenario is slightly better aligned with expected changes in annual water yield patterns than the *country* scenario. In 2000, average annual water yield on all rainfed cropland grid cells across the globe was estimated to be 636 mm km^−2^. In 2015, the average yield is projected to be 645 mm km^−2^ under the *regional* scenario and 632 mm km^−2^ under the *country* scenario. Maps of expected changes in water availability on rainfed cropland around the globe and for two regions of the world across the two scenarios are shown in Figures 4 and 5.

### Biomass carbon emissions due to land conversion

**Figure 6:**
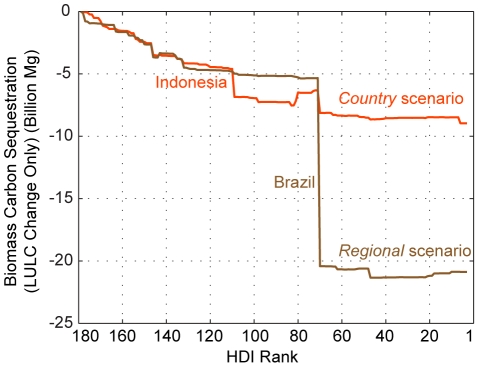
Projected net change in global biomass carbon storage (sequestration) due to land conversion. Country-level contribution to the projected global change in biomass carbon storage (sequestration) is sorted by HDI rank. The portions of the curve that increase indicate countries expected to experience a net gain in storage due to LULC change. Countries projected to experience significant changes are noted. This graphic does not include the 0.0008 and 0.0005 billion Mg net loss in biomass carbon storage due to land conversion in unclassified HDI countries under the *country* and *regional* scenarios, respectively.

Cumulative country-level changes in biomass carbon storage due to land conversion is sorted by HDI rank (Figure 6). Most of the difference between the two scenarios is explained by the projected loss of broadleaved forest area in Brazil (Figure S1). Under the *regional* scenario, 0.84 million km^2^ of additional broadleaved forest are lost between 2000 and 2015 in Brazil when compared to *country* scenario. Other than Brazil, most of the net loss in biomass carbon occurs in countries with HDI ranks of less than 100 (the least-developed countries). Some countries with high HDI show a net gain in biomass carbon storage because their cropland abandonment rates are close to or even outpace their urban growth rates.

### Loss in undeveloped land and species persistence

**Figure 7:**
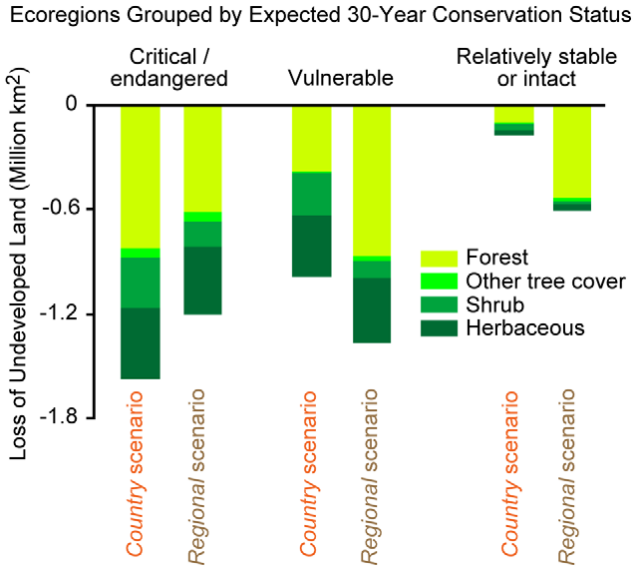
Projected global change in undeveloped land area from 2000 to 2015 by ecoregion status. Each bar indicates the loss of undeveloped land area by vegetation type [109]. See the text and [84] for more information on ecoregion status. In this graph we do not include any undeveloped land area provided by cropland abandonment from 2000 to 2015.

Global cropland and urban area expansion reduces the global supply of undeveloped land (non-urban and non-cropland covers), a land type that is more likely to provide species habitat than other land uses. In Figure 7 we summarize the gross conversion of global undeveloped grid cell area by ecoregion conservation status and scenario (by gross we mean that we do not include cropland that is abandoned to less intensive uses). An ecoregion's conservation status indicates the degree of habitat alternation and spatial pattern of remaining habitat in an ecoregion at the end of the 20^th^ century [84]. Critical and endangered ecoregions retain little natural habitat and the habitat that remains is highly fragmented and the continued persistence of many species is highly uncertain. Vulnerable and relatively stable ecoregions are less disturbed. While the *regional* scenario converts more undeveloped grid cell area over the 15 year period (3.2 versus 2.7 million km^2^), the *country* scenario converts more undeveloped grid cell area in the most endangered ecoregions (1.6 versus 1.2 million km^2^ in critical/endangered ecoregions).

**Figure 8:**
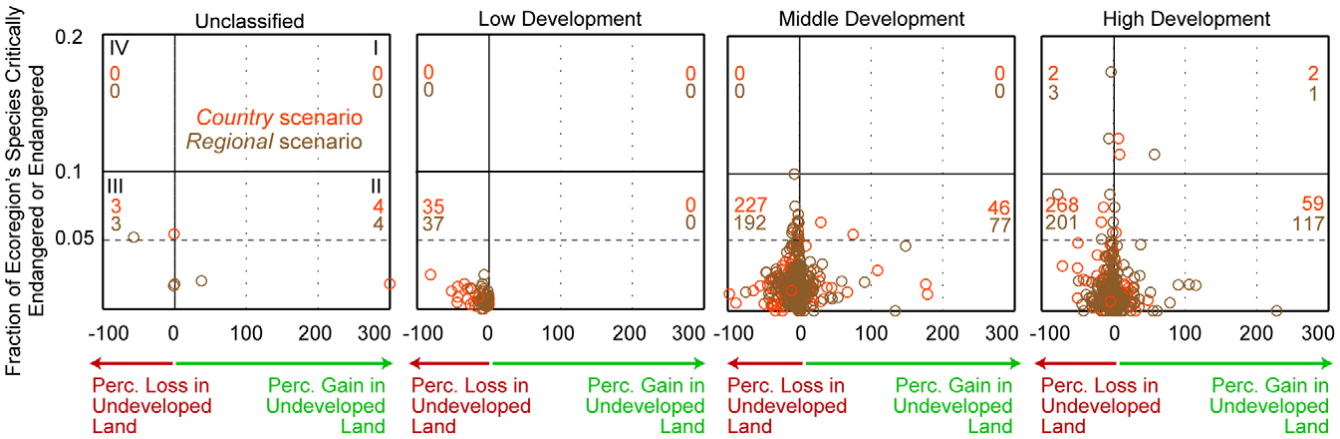
Projected global change in undeveloped land area versus species endangerment status by ecoregion. Each circle represents an ecoregion. The two numbers in each quadrant (defined by 0.1 on the *y*-axis and 0 on the *x*-axis) in each graph indicate the number of ecoregions in a quadrant under the *country* and *regional* scenarios. Change in undeveloped land area is a net measure because we assume abandoned cropland that does not convert to urban area becomes undeveloped land. Data on an ecoregion's fraction of critically endangered and endangered species comes from [75].

We also plot each ecoregion's net relative change in undeveloped grid cell area versus the percentage of the ecoregion's species that are critically endangered/endangered according to the IUCN Red List by HDI category [75], [85] (Figure 8). An ecoregion can experience a net grid cell area increase in undeveloped land if its growth in abandoned cropland grid cell area (not including the cropland abandoned to urban use) is greater than its loss of undeveloped land to urban and cropland grid cell area [86]. While all ecoregions in low development countries show net loses in undeveloped land across both scenarios, none of these ecoregions are particularly rich in critically endangered/endangered species when compared to some ecoregions in the middle and high development countries. Finally, Figure 8 indicates that many more ecoregions in middle and high development countries are projected to experience a net loss in undeveloped grid cell area under the *country* scenario than under the *regional* scenario. However, particularly large losses of undeveloped land in the Madeira-Tapajós moist forests (Amazon Basin), Southwest Amazon moist forests, Uatuma-Trombetas moist forests (Amazon Basin), and Kazakh steppe ecoregions under the *regional* scenario account for that scenario's greater conversion of undeveloped land around the world as indicated by Figure 7.

### Avoided emissions analysis

**Figure 9:**
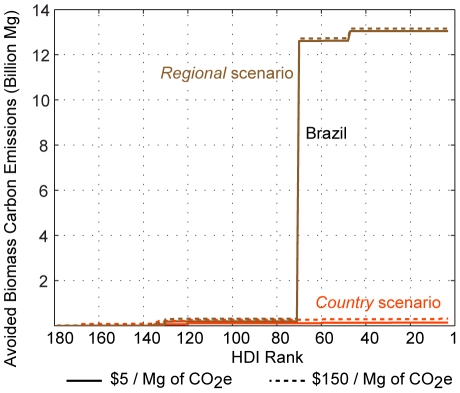
Projected avoided biomass carbon emissions due to an avoided emissions program. Expected country-level contribution to avoided global biomass carbon emissions at offset credit prices of $5 and $150 Mg^−1^ of CO_2_-e is sorted by HDI rank. Brazil's contribution is noted on the graph.

In Figure 9 we summarize avoided emissions assuming a REDD-like program existed as of 2000 and that our scenarios were used by program administrators to determine business-as-usual deforestation and associated carbon emissions rates. We determine avoided emissions for two avoided deforestation credit prices, $5 and $150 Mg^−1^ per emissions of CO_2_e (carbon dioxide-equivalent) avoided. The *regional* scenario, which had more than twice the loss of stored biomass carbon than the *country* scenario, generates far more avoided emission credits. In this illustration, avoided emissions supply is barely affected by offset price; $5 offsets generate almost as much avoided emissions as do $150 offsets. This result occurs because the net returns to agriculture are, on average, very low in Brazil, the source of most credits under both scenarios. This suggests that, assuming transaction and other program costs are kept low, modestly priced carbon offsets could prevent an aggressive acceleration in agricultural development in Brazil.

### Tradeoffs

To summarize, we observe a tradeoff among LULC change and ecosystem service and species habitat provision from 2000 to 2015. The tradeoff is less severe under the *country* scenario. Specifically, the grams of carbon released due to LULC conversion per additional calorie of crop produced is 62% less and the loss of undeveloped area (our proxy for species habitat) per additional calorie of crop produced is 24% less under the *country* scenario when compared to the *regional* scenario. The *country* scenario's more efficient production of calories relies heavily on a 1) fairly dramatic expansion in irrigation capacity, 2) greater cropland expansion in countries with greater access to technological improvements in agriculture, and 3) avoidance of large-scale land conversion in important biomass carbon storage areas. The *regional* scenario outperforms the *country* scenario on only one modeled metric: rainfed cropland under the *country* scenario is expected to receive less water than it is under the *regional* scenario. Therefore, if 1) the relatively heavy expansion in irrigation capacity that underpins the *country* scenario's results does not occur as modeled and 2) the greater water shortages on rainfed cropland under the *country* scenario significantly impairs rainfed cropland productivity then the tradeoff gap between the two scenarios would shrink.

## Discussion

In this paper we demonstrate a straightforward method for allocating expected LULC change given at a spatially coarse level to a grid cell-level and predicting the impacts of such mapped change on the provision of several ecosystem services and habitat (see Text S1 for a comparison of projected changes to actual changes that have occurred since 2000). Our spatial allocation method is a cellular process that is guided by maps that describe how well-suited each grid cell is to a particular land use. We then use the InVEST methodology to translate LULC changes into changes in the provision of various ecosystem services and undeveloped land (our proxy for species habitat). This approach is transparent and well suited to cases where data and technical expertise are limited.

In our illustration of this approach we are temporally modest. We only project to 2015 for several reasons. First, there are global projections for regional agricultural land use and grid cell-level population density out to 2015. Further, extrapolating country-level trends in cropland over a 20-year period (the mid 1990s to the mid 2010s) seems reasonable. Second, by only projecting to 2015 we minimize the bias in our models caused by our present inability to more comprehensively incorporate impacts of climate change and other dynamic feedback effects (see below). However, we feel our approach is also well-suited for the exploration of the global impacts of much more distant future scenarios. In such analyses, expectations for country- or regional-level land-use change would not be based on well-calibrated models' projections for the near future but on plausible global change trajectories. For example, what would the pattern and magnitude of environmental impact be across the globe if the developing world adopted the current diet preferences of the developed world by 2060? It has been estimated that such a future would require an additional 26 million km^2^ of cropland compared to year 2000 levels (approximately the combined area of Canada and Russia; *personal communication* with David Tilman). Using various plausible cropland suitability maps and cellular allocation processes consistent with basic economic theory and broad expectations for changes in climate, technology, population and consumer preferences, we could create several global maps of LULC that might meet such a goal and calculate each projection's impact on ecosystem service and habitat provision. For example, one alternative cropland suitability layer in this analysis could relax the restriction on cropland development in protected areas according to data on the effectiveness of protected area management [87]. Identification of the preferred cropland development path and the policy levers necessary to get to the preferred change path could follow.

This potential for our methodology to inform land-use and ecosystem service provision policies, either in the short or long-term, is particularly exciting. For example, cropland suitability scores could be increased in grid cells in parts of the world that governments have targeted for cropland expansion or agricultural subsidies. The ecosystem service ramifications of these spatially-explicit policies could then be estimated and compared to business-as-usual development patters. In this paper we illustrate how one could use LULC change projections to establish a baseline of deforestation to set eligibility and cap requirements in a global REDD-like program instead of relying on historic deforestation rates [88]. Using projected LULC maps instead of historic deforestation rates to guide a global REDD-like program avoids the perverse result of rewarding countries that aggressively deforested in the immediate past. Finally, as noted above, policy makers could use this tool to begin a discussion on the more distant future and the impact that another 50 or 100 years of development might have on the earth's environment.

### Potential improvements in our methodology

The general approach described here can be improved in a number of ways. As of 2000, approximately 12% of the earth's land surface was used for cultivation while 22% was used for pastures and rangelands [89]. Growing global demand for meat would suggest additional forest and natural grasslands will be converted to pasture and rangeland, especially in Africa and Latin America [90]. We do not currently account for such land conversion in our scenarios, primarily due to inadequate data on current and expected pasture land area in many regions of the world. Including pasture land as an explicit LULC category, modeling its change, and its impact on ecosystem services and habitat would no doubt improve our general approach. However, we do not believe that ignoring pasture change significantly compromises our general approach, especially given the world's increasing reliance on confined livestock production (in 2003, 70% of developed countries' consumption of meat, milk, and eggs came from livestock largely raised in confined settings) [90, *personal communication* with David Tilman]. Confined livestock production is largely supported by grains, not pasture. This suggests that most new agricultural land around the globe in the future will be devoted to crop production rather than grass production.

The lack of pasture land in our model is not the only LULC change dynamic missing from our approach. In fact, any change in LULC that does not involve urban or cropland area is ignored (e.g., we do not account for forest to wetland transitions). Further, for the types of LULC change we do account for, we do not measure and spatially allocate all change, just the change necessary to spatially allocate net change. Therefore, our approach underestimates the amount and variety of LULC change around the world. Data limitations explain the absence of these LULC change dynamics from our approach. To illustrate our approach's limitations and the impact of these assumptions consider LULC change in the United States from 1992 to 2001. During that time period the US experienced a 400 km^2^ net gain in agricultural area (cropland, pasture, and rangeland area). In our approach we would have only worked with the cropland portion of this net change. However, assume our approach was modified to model change in agricultural area, not just cropland area. In our spatial allocation approach, after allocating the 7,200 km^2^ of urban area that emerged from cropland, pasture, and rangeland from 1992 to 2001, we would have spatially allocated 7,600 km^2^ of new cropland, pasture, and rangeland area over forest, wetland and grassland area to account for the net gain of 400 km^2^. However, during this 1992 to 2001 period 17,300 km^2^ of agricultural land was converted to forests, wetlands and grasslands; a LULC change dynamic our approach does not account for [91]. Therefore, if we were to model all LULC change, the 400 km^2^ net gain in agricultural land from 1992 to 2001 would include the conversion of 24,900 km^2^ of grassland, wetlands, and forests to agricultural uses and not just the 7,600 km^2^ we would have modeled (24,900 km^2^ less the 7,200 km^2^ of urban area that emerged from agricultural use and the 17,300 km^2^ of agricultural lands lost to other uses generates a net gain of 400 km^2^).

The approach in this paper uses potential cereal yield under intensive management as a proxy for cropland suitability. Suitability layers that incorporate regional crop and crop production mixes could be developed. These more nuanced layers could also incorporate other factors that affect crop and management choice including proximity to markets and transportation networks, local crop prices, local policies and land tenure issues, production costs, and consideration of crop failure risks [4], [74], [92]–[97]. The proper combination of all of this data would generate suitability layers that measure the expected net revenues from farming in each grid cell. Such net revenue layers would improve our analysis in several respects. First, it would lead to more accurate predictions of where crops would be grown and therefore, the effect of cropland expansion on ecosystem service and habitat provision. As we have shown here the spatial allocation of cropland can have a major effect on biomass carbon emissions and habitat provision and also significantly impact water quality, soil conservation, and nutrient cycles [5], [93], [98]. Second, such suitability layers would improve the analyses of policies that are a function of land-based opportunity cost. For example, in our avoided emission analysis we assign a country's average net revenue from cropland to each cropland grid cell in the country instead of grid cell-specific estimates. Using one estimate of cropland net revenues across a whole country means for a given carbon credit price that either all or none of the eligible grid cells in the country will avoid deforestation (“bang-bang” solutions). In reality, the forested areas that would generate the lowest returns in cropland as of 2015, the areas where payments for avoided deforestation would make the most economic sense, are scattered across the globe. A lack of data prevents us from creating a cropland suitability map based on net revenues. While crop yield and basic agriculture management data from across the globe are becoming more readily available, globally available data on production and related costs, country-level policies and land tenure issues, and crop production uncertainties are lacking [99], [100].

In this paper we only use a few of the InVEST ecosystem service models and the ones that are presented are the simplest versions available. For example, InVEST includes water quality, soil conservation, and agricultural greenhouse gas emissions models that could be applied to our scenarios. However, to use these models we would first have to create global maps of chemical use and land-management. Further, with better data we could have used more sophisticated InVEST models in this analysis. For example, in the simple habitat model used here we assume all undeveloped area provide habitat of some sort to species. More precisely, by aggregating all undeveloped area in an ecoregion we are implicitly assuming that undeveloped land provides habitat of the same quality to all species. However, habitat closer to urban areas and croplands may not be able to support species to the same degree than more isolated habitat can [101]. Further, not all undeveloped land will be suitable to all species; a suitable cover for one species may not be usable by another. InVEST contains a model that allows us to adjust habitat quality based on proximity to disturbances and ecosystem type. However, this analysis requires additional work to identify and map threats and habitat preferences of species guilds by ecoregion. Finally, as an alternative to InVEST, we could have combined our predictions of global LULC change with more complex water models and carbon models such as SWAT and CENTURY [102]–[104]. These models, however, tend to require sophisticated users and detailed data and are designed to work at the regional scale; these characteristics will likely restrict their applicability with more general analyses and in many regions.

Another important limitation in our analysis, and almost all current work on LULC and ecosystem services and habitat provision change, is the lack of adequate treatment of dynamic feedbacks and thresholds [56]. For example, our model does not consider how cropland pattern and use will respond to climate change, changes in availability of inputs, or changes in technology. Farmers could adapt to changes in climate by changing crop choice, improving input management, adopting water saving technologies such as drip irrigation, or simply relocating. Such changes, however, may take many years and involve large sunk costs and significant learning [105], [106]. Changes in climate will also affect urban development patterns [107]. Potential threshold events associated with climate change are also not included. Further, we do not consider technological or biophysical thresholds that may create abrupt changes in service provision by ecosystems. For example nutrient loadings may cause estuaries and freshwater systems to undergo abrupt change [108]. By projecting LULC change and associated impacts on ecosystem service delivery to 2015 we minimize the bias in our models caused by our present inability to more comprehensively incorporate impacts of climate change and other dynamic feedback effects.

## Materials and Methods

### Spatially allocating 2000 to 2015 urbanization

We begin with a gridded map of the globe from 2000 where each grid cell is assigned a LULC (the cells are 5 km×5 km at the equator, see Text S1; [109], [110]). In each country we convert grid cells into urban use such that the projected 2000 to 2015 increase in urban grid cell area in the country is met [8]. Let 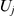, 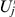, and 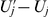 indicate grid cell area in urban use in country *j* in 2000, grid cell area in urban use in country *j* in 2015, and net change in urban area in country *j* from 2000 to 2015, respectively (Table S2). 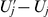 is never less than 0.

To spatially allocate 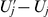 we use an urban suitability map and a land-use change simulation module for the Idrisi Andes geographic information system called GEOMOD [58], [59]. in the change simulation GEOMOD tends to convert grid cells that have higher urban suitability scores and are in close proximity to existing urban grid cells into urban use. A GEOMOD simulation runs in annual time steps such that a portion of 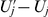 is allocated each year from 2000 to 2015 until all of 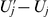 has been allocated by 2015. We assume grid cells that are projected to have higher 2015 population densities [60] and that are flatter [61] are the most suitable for urban use. Specifically, grid cells with slopes of less than 12% receive values of 5, between 12 and 18% receive values of 4, between 18 and 30% receive values of 3, between 30 and 50% receive values of 2, and 50% or greater receive values of 1. Further, grid cells with a predicted 2015 population density of zero receive values of 1 and values greater than zero receive values ranging from 2–5 using an equal interval system where areas with higher predicted densities are given scores closer to five. A grid cell's suitability score is the average of its scores on the two metrics. GEOMOD uses suitability layers on a scale of 0 to 100. Therefore the program converts the urban suitability map from the 1 to 5 scale to a 0 to 100 scale before it runs the land-use change simulation (Figure S2). Grid cells that are in protected areas as of 2000 (IUCN categories of I through VI category) are not eligible for urban land use in 2015 [62]. See Text S1 for more information on the calculation of 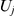, 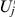, and 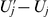 and the spatial allocation of 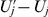.

### Cropland grid cells, cropland area, and harvested area in 2000

We designate each grid cell on the 2000 LULC map in the covers “Cultivated and managed areas”, “Mosaic: Cropland/Tree Cover/Other natural vegetation”, and “Mosaic: Cropland/Shrub or Grass Cover” as cropland [109], [110]. We use the term **cropland grid cell area** to refer to the aggregate area of grid cells designated as cropland. Let *C_j_* and *C_r_* indicate the cropland grid cell area in country *j* and region *r*, respectively, on the 2000 LULC map. There are 25.4 million km^2^ of cropland grid cell area on the 2000 global LULC map. As suggested by the cover names that we collectively refer to as cropland, these cells can contain a mix of other LULC, including pasture, roads, villages, etc. Hereafter we use the term **cropland area** to refer to the area in cropland grid cells actually used to grow crops. (Cropland area includes land under temporary crops (double-cropped areas are counted only once), temporary meadows for mowing or pasture, land under market and kitchen gardens and land temporarily fallow. The abandoned land resulting from shifting cultivation is not included. This estimate also includes crops that occupy the land for some years and need not be replanted after each annual harvest, such as cocoa, coffee and rubber. This category includes flowering shrubs, fruit trees, nut trees and vines, but excludes trees grown for wood or timber.)

In any given year not all cropland area produces cropped output because of fallow practices and crop failures. Conversely, some cropped areas are used multiple times in a calendar year. Let the area of crop production used over the course of a year be known as **harvested area**. Let *H_j_* and *H_r_* indicate the estimate of harvested area in 2000 in country *j* and region *r*, respectively where croplands that produced crops multiple times in 2000 are double or triple counted (e.g., if *X* km^2^ of cropland in a country produced one crop in 2000 and if *Y* km^2^ of cropland in the country produced two crops in 2000 then the country's harvested hectares in 2000 are *X*+2*Y*) [6].

### Calculating and spatially allocating net change in cropland grid cell area

We estimate changes in cropland area around the globe between 2000 and 2015 twice. In the *country* scenario we extrapolate 1985 to 2000 cropland area change rates at the country-level out to 2015 [6]. In the *regional* scenario we use [7] 's predictions of regional-level 2015 cropland area for major crops to determine rates of cropland area change at the region-level (see Table S3 for a list of countries by region). Let the rate of change in cropland area from 2000 to 2015 in country *j* or region *r* be given by 
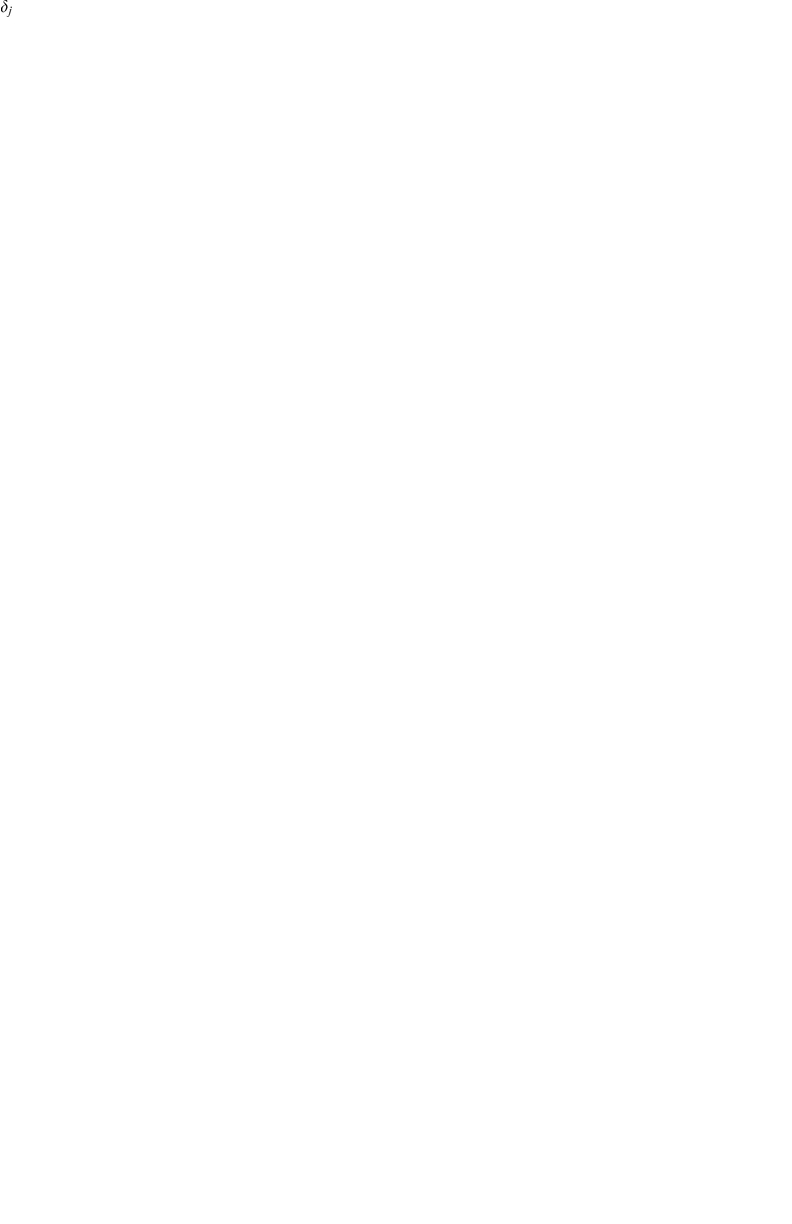
 or 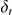 where 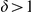 (<1) indicates a net increase (a net decrease) in cropland area from 2000 to 2015.

The cropland grid cell area in country *j* and region *r* in 2015 under the *country* and *regional* scenarios are given by 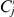 and 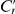, respectively,
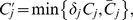
(1)

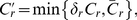
(2)where 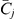 and 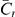 are *j*'s and *r*'s total arable grid cell area, respectively (Michael Jennings, *personal communication*). Arable area does not include urban grid cells in 2000 and 2015 and grid cells in protected areas as of 2000. By using equations (1) and (2) to project 2015 cropland grid cell area we assume that a country or region's cropland grid cell area will expand (or contract) at the same rate as the country or region's growth in cropland area. In other words, we assume that, on average, the density of cropland area within a country or region's cropland grid cell area is the same in 2015 as it was in 2000.

If a country or region is expected to experience a net gain in cropland grid cell area between 2000 and 2015, we decompose 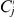 and 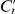 into cropland grid cell area established between 2000 and 2015, 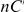, and cropland grid cell area established before 2000 but remains on the land as of 2015, 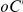, with the following system of equations,
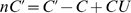
(3)

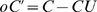
(4)

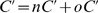
(5)where *CU* is the cropland grid cell area as of 2000 (*C*) that converted to urban land between 2000 and 2015 in country *j* or region *r*. Otherwise, if a country or region is expected to experience a net decrease in cropland grid cell area between 2000 and 2015 then *C* –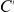 gives the net cropland grid cell area lost between 2000 and 2015. In such cases 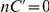 and 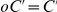 if 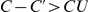 or 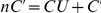 and 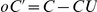 otherwise. Lost cropland grid cells either convert to urban use or become abandoned cropland with landcover equal to the dominant vegetation cover in the cell's ecoregion.

Using GEOMOD and the cropland suitability map we spatially allocate 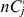 from the *country* scenario in each *j* given the already allocated expansion in urban grid cells. Again this is done in annual time steps such that all 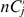 grid cells are allocated by 2015. Cells in urban use in 2000 or 2015, not in *j*'s arable land zone, or in protected areas are not available for cropland use in 2015. If county *j* is projected to experience a loss in cropland grid cell area beyond that which is converted to urban use, then cropland cells with the lowest cropland suitability scores tend to be removed from the landscape by the simulation method. See Table S4 for *country* scenario results. We repeat the cropland grid cell allocation process on the global map with already allocated expansion in urban grid cells for each region under the *regional* scenario (Text S1, see Tables S5–S6). After all of this we have two global 2015 LULC maps where the pattern of urbanization on both maps is the same and grid cells that did not urbanize or convert to or from cropland remain in their 2000 LULC.

The cropland suitability map is a function of slope [61] and potential cereal yield under intensive management [63] where grid cells with gentler slopes and higher potential yield are given higher suitability scores. When constructing the potential yield map we had to choose between the rainfed and irrigated potential cereal yield in each cell. We assign the irrigated yield to the cells in *j* that have potential yield characteristics that are correlated with the potential yield characteristics of grid cells with high irrigation use in *j* as of 2000 (as measured by percentage of cell irrigated; 18% of global cropland (2.79 million km^2^) was equipped for irrigation in 2000; see [73], Text S1, Figure S3, Tables S7–S8). In other words, the irrigation model tends to select the irrigated potential yield for cells that were highly irrigated as of 2000 and those that were not cropland as of 2000 but had potential yield combinations that were very similar to those that were highly irrigated in 2000. A cell's suitability score was the average of its slope and potential yield scores. As before, GEOMOD converted suitability scores from a 1 to 5 scale to a 0 to 100 scale before simulating change (Text S1, Figures S4–S5).

### Calculating harvested area in 2015

Next we convert 2015 cropland grid cell area in a country into 2015 harvested area in the country. Expected harvested area in 2015 in country *j* is given by 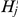,
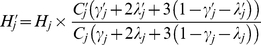
(6)where 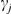 gives the fraction of *j*'s grid cell cropland area in a single cropping zone in 2000, 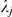 gives the fraction of *j*'s grid cell cropland area in a double cropping zone in 2000, and 
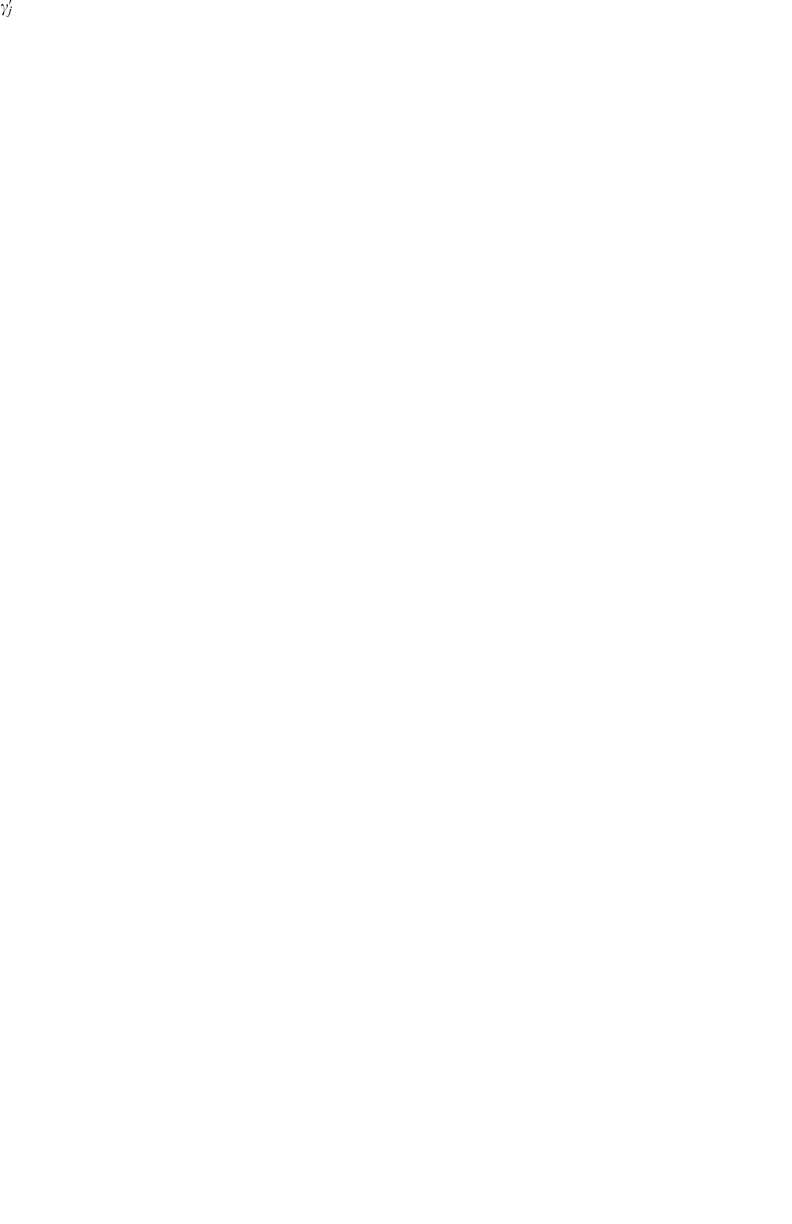
 and 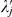 indicate the fraction of 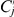 in single and double cropping zones in country *j* in 2015 ([63], Text S1, and Table S9). We calculate 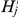 twice for each *j*, once under the *country* scenario and another time under the *regional* scenario (before using equation (6) with *regional* scenario estimates we convert 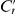, 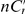, and 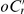 into 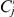, 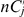, and 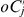 values). In equation (6) we assume that the harvested area in *j* expands at the same rate as the multi-cropping-weighted growth of cropland grid cell area in *j*. This means we assume that, on average, crop failure rates, fallow management, and cropping behavior in multiple cropping zones in a country will be the same in 2015 as it was in the country in 2000.

The harvested area established after 2000 in country *j* under a scenario is given by,
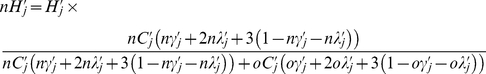
(7)where the ‘*n*’ and ‘*o*’ in front of the Greek letters indicates the share of 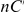 and 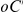, respectively, in the various cropping zones in 2015. Finally, harvested area in country *j* established prior to 2000 is given by,
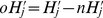
(8)


We calculate 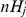 and 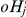 twice for each *j*, once under the *country* scenario and another time under the *regional* scenario.

### Measuring change in crop production between 2000 and 2015

To determine 2015 crop production in country *j* we first calculate each crop or crop types average yield in country *j* in 2000, given by *Y_ji_* and measured in in Mg ha^−1^, where *i* = 1, 2, …, *I* includes individual crop types (e.g., rice) or crop groups (e.g., treenuts; [6], Text S1, and Table S10). For example, in 2000 96,000 hectares of maize and 124,000 hectares of barley were grown in Afghanistan; both crops belong to the coarse grains category (so do sorghum and oats, however, neither were produced in Afghanistan in 2000 according to [6]). Respective production was 114,998 Mg of maize and 73,991 Mg of barley. Therefore, coarse grain yield in Afghanistan in 2000 was 0.859 Mg ha^−1^ (i.e., 114,998 Mg + 73,991 Mg/96,000 ha +124,000 ha).

We determine the caloric production of crop type *i* in *j* in 2015 with,
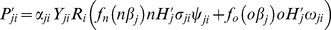
(9)where 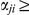
1 is country *j*'s expected 2000 to 2015 growth in *i*'s yield due to technological change ([7], [65], Text S1, and Table S11); *R_i_* is the average caloric content Mg^−1^ of crop or crop group *i* ([82], Text S1, and Table S12); 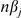 and 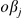 indicate the relative difference in the cropland productivity of the new and old cropland grid cells in 2015, respectively, versus the cropland grid cells in *j* as of 2000, the functions *f_n_* and *f_o_* convert 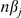 and 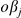 into yield inflators; 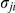 indicates the fraction of “new” harvested hectares in *j* in crop or crop group *i* in 2015, 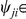 [0,1] indicates the degree to which new harvested hectares used for crop or crop group *i* in *j* meets its projected productive capacity by 2015 where a 1 indicates it has reached its projected productive capacity; 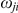 indicates the fraction of “old” harvested hectares in *j* that is in crop or crop group *i* in 2015; and 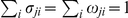. The first term in equation (9), 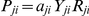, is an estimate of year 2000 ha^−1^ caloric production of crop or crop group *i* in country *j* and the second term converts this to 2015 levels given technology growth, changes in overall productivity of the grid cells used for crops, and crop mix changes. Relative changes in productivity, 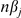 and 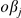, are given by relative changes in the suitability of cropland grid cell area in *j*. Specifically, the relative difference in the productivity of cropland grid cell area in 2015 versus 2000 in country *j* is found by dividing the 2015 average suitability score for new or old cropland grid cell area in *j* by the average suitability score for *j*'s cropland cell area in 2000 (Text S1 and Table S13). For example, if the average cropland suitability score for new cropland grid cells in country *j* is 55 and the average agriculture suitability score for cropland grid cells in country *j* on the 2000 map is 49 then 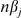 = 55/49 = 1.12. For illustrative purposes we set 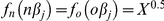 for all *j* under both scenarios. The value of 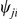 will depend on the crop or crop group, the land used for *i* in *j*, the technology applied to production for *i* in *j*, and agricultural-related infrastructure that can be accessed by the newly converted cropland in country *j*. Some lands will produce expected yields within a year or two of establishment while others may take a decade or more. Due to a lack of data we set 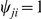 in our model for all combinations of *j* and *i*. If we drop *R_i_* from equation (9) then 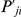 gives 2015 country-level production of crop or crop group *i* in metric tons. The change in the production of crop type *i* in *j* from 2000 to 2015 is given by 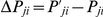 and the change in all crop production in country *j* is given by 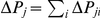.

We create 5 allocations of 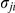 across all combinations of *j* and *i*. Let 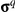 indicate the *q* ^th^ matrix of 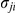 values. One realization of 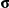 indicates the relative mix of crop types in each *j* as observed in 2000 (*q* = Year 2000 crop mix; [6], Text S1, and Table S14). In another projection we assume that the areal share of crop types other than rice, wheat, coarse grains, and oil seeds in each country will fall steeply by 2015 when compared to 2000 levels (*q* = 2; Text S1 and Table S15). In another we assume that the areal share of crop types other than rice, wheat, coarse grains, and oil seeds in each country will fall moderately by 2015 when compared to 2000 levels (*q* = 3; Text S1 and Table S16). In another we assume that the areal share of all crop types in each country will remain close to 2000 levels (*q* = 4; Text S1 and Table S17). In the last projection we set crop share values across countries such that the global area devoted to rice, wheat, coarse grains, and oil seeds matches that predicted by the agricultural trade model in [7] (*q* = Projected 2015 crop mix; [7], Text S1, and Table S18). In this illustration we set 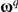
equal to 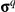; there is no particular reason, however, why these must be equal.

Every input into equation (9) can be described with uncertainty. For example, for crop groups *i* we only use average yields and do not consider the observed distribution's other moments. Moreover, we do not consider how *Y_ji_* of crop groups *i* will change over time due to changes in the areal mix of crops within the group. Nor do we consider how *Y_ji_* might change as the climate changes, etc. Further, we assume that the effect of 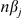 and 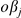 on yields are given by the functional form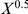; other functional forms are not experimented with. In addition, the values of 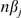 and 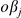 will be sensitive to variables other than the relative change in cropland suitability, including the specific allocation of crop types across *j* and water availability. The expected paths of agricultural technology development 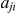 are also highly uncertain. We experiment a bit with alternative crop choice allocations, although the matrices 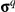 and 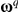 could be perturbed even more.

### Annual water yield

Annual water yield, measured in mm km^−2^, is equal to the precipitation that falls during the course of a year less the water that evaporates or transpires (actual evapotranspiration) [2], [67]–[72].

Annual water yield in grid cell *x* In LULC *j* in 2000 is given by *W_xj_* and in 2015 by 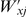. Hereafter we describe how to calculate *W_xj_*. The variable 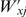 is calculated in the same manner but with 2015 data, including LULC cover in grid cell *x* and precipitation in grid cell *x*. Let,
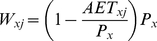
(10)where *AET_xj_* is the annual actual evapotranspiration on grid cell *x* with LULC *j* in 2000 (measured in mm km^−2^) and *P_x_* is the year 2000 precipitation on grid cell *x* (measured in mm km^−2^). Therefore, *W_xj_* represents the amount of water available to the surface and groundwater systems in *x*'s watershed over the course of 2000.

The portion of equation (10) that represents evapotranspiration, *AET_x_*/*P_x_*, is an approximation of the Budyko curve,
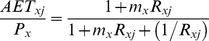
(11)where *m*_x_ is the dimensionless ratio of plant accessible water storage to expected precipitation during the year and *R_xj_* is the dimensionless Budyko Dryness index on grid cell *x* in 2000 and is defined as the ratio of potential evapotranspiration to precipitation [68], [70].

As defined by [70], *m*_x_ is a non-physical parameter that characterizes the natural climatic-soil properties in grid cell *x* [69], [71], [72],
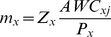
(12)where *AWC_xj_* is the volumetric (mm) plant available water content on grid cell *x* in LULC *j* [111], a soil property generally estimated as the difference in water content (mm) between field capacity and wilting point (i.e., the amount of water that can be held and released in the soil for use by a plant), and *Z_x_* is a constant that is calibrated for climatically homogeneous basins (i.e., *Z* is the same for all grid cells in a climatically homogeneous basins). The soil texture and effective soil depth in grid cell *x* defines *AWC_x_*. The constant *Z* adjusts the water balance to account for timing differences of monthly intra-annual rainfall and energy distribution patterns, and rainfall intensities. Variation in *m* across a landscape reflects variation in available water content and rainfall across the landscape. Although we recognize that the relationship between *w* and *AWC*/*P* is technically non-linear, we assume a linear relationship to simplify modeling.

We define the Budyko dryness index as follows, 
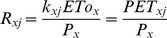
(13)where *ETo_x_* is the reference evapotranspiration on grid cell *x* [112], [113] and *k_xj_* is the plant evapotranspiration coefficient associated with the LULC *j* on grid cell *x*. *ETo* is an index of climatic demand while *k_xj_* is largely determined by *j*'s vegetative characteristics [114]. Budyko dryness index values that are greater than one denote grid cells that are potentially arid [68].

We use the HadCM3 climate model with the Intergovernmental Panel on Climate Change A2 greenhouse-gas emissions scenario to map global annual precipitation [66]. Because global maps of precipitation under HadCM3 with the A2 emissions scenario are not available for 2000 and 2015 we use a modeled precipitation map from 1990 with the 2000 LULC map and a modeled precipitation map from 2025 with the 2015 LULC maps. Therefore, depending on the pace of climate change, our grid cell-level changes in annual water yield will deviate slightly from estimates we would have generated if we had been able to use 2000 and 2015 precipitation maps with the 2000 and 2015 LULC maps.

See Table S19 for data on average country-level water yield on rainfed cropland grid cells in 2000 and 2015 under both scenarios.

In our analysis we imply that a decline in average annual water yield in an area means that rainfed cropland yields in the area may not increase at the rate of technological growth, all else being equal (in addition, less runoff will be available for irrigation use). However, this may not be the case in a given area for several reasons. First, if an annual reduction in water yield in an area is primarily due to reductions during the non-growing season then rainfed crop production may not be affected by a decrease in annual water yield (unless the changes in the off-season affect water storage and soil productivity in the growing seasons). Second, even if water availability decreases during the growing season farmers can adapt by changing planting patterns or management such that productivity does not decline [115]. (Others have argued, however, because farmer adaptation to climate change may take many years and involve large sunk costs [106], a change in water yield on rainfed cropland will have, at least in the short run, a negative affect on productivity.)

### Change in aboveground biomass carbon storage

Let *Z_j_* indicate the total mass of aboveground and belowground biomass carbon stored as of 2000 in country *j*'s grid cells that convert to urban or cropland use by 2015 [116]. Let 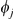, 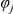, and 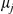 indicate the average Mg ha^−1^ of biomass carbon stored in *j*'s urban, cropland, and less-intensely managed grid cells as of 2000, respectively, where less-intensely managed grid cells are any non-cropland and non-urban grid cells ([116] and Text S1). The change in biomass carbon in country *j* from 2000 to 2015, measured in metric tons, due to land conversion only, is given by,
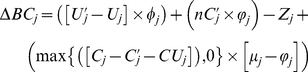
(14)


The first term of equation (14) gives the expected biomass carbon storage as of 2015 in cells that convert to urban use in country *j* (assuming that new urban areas have attained the average storage values of urban areas that existed as of 2000). The second term gives the expected aggregate biomass carbon storage in cells that convert to cropland use in country *j* (assuming that new urban areas have attained the average storage values of urban areas that existed as of 2000) The last term gives the expected aggregate biomass carbon storage in abandoned cropland grid cells (that do not convert to urban use) less the biomass carbon stored in the land when it was in cropland in 2000 (assuming that abandoned cropland areas have attained the average storage values of less-intensely managed areas that existed as of 2000). Recall that we do assign a particular vegetation cover to abandoned cropland. However, we do not use the specific biomass carbon storage values associated with these covers in equation (14); instead we use a country-wide average storage value associated with all less-intensely managed types. We do this because resulting vegetative cover on abandoned cropland is highly speculative on our part and using the average value across all less-intensely managed types is more conservative.

The metric 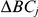 does not account for 1) carbon accumulation in grid cells that do not experience a LULC change (e.g., the sequestration from 2000 to 2015 in forests that mature over that period); 2) land management activities that may affect sequestration rates on converted land; and 3) the affect of climate change on storage capacities and sequestration rates (ha^−1^ storage estimates are from circa 2000). See Figure S6 for maps of 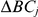.

### Avoided emissions analysis

The first step in this analysis is to identify which nations would have been eligible to generate avoided emission credits from 2000 to 2015. Avoided emission credits will only be given to developing nations that are predicted to experience deforestation rates greater than historic rates. Let *D_j_* represent the predicted annualized deforestation rate from 2000 to 2015 in country *j* where,
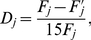
(15)


*F_j_* is the forested area in country *j* in 2000, and 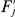 is a scenario's forested area in *j* in 2015. Let *D_jb_* indicate the observed per annum 1990 to 2000 deforestation rate in country *j* [77]. A developing country would have been eligible to begin generating avoided emissions credits beginning in 2000 if *D_j_*>*D_jb_*.

We assume the value of urban land is always larger than any potential value of an offset. Therefore, an eligible country would have only generated avoided emission credits through avoided conversion of forest to cropland. The maximum number of credits that a country can generate from avoiding forest conversion to cropland is equal to the predicted biomass carbon emissions between 2000 and 2015 due to deforestation for cropland that is above and beyond the historic deforestation baseline rate, or,
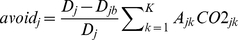
(16)where *A_jk_* are the hectares of forest type *k* in country *j* in 2000 that are projected to be in cropland by 2015 under a scenario, *A_j_* = 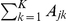, *CO2_jk_* is the average CO_2_-equivalent (CO_2_e) of carbon stored in biomass ha^−1^ of forest type *k* in *j* in 2000 ([116], Text S1, and Table S20). The first term in equation (16) gives *j*'s share of emissions due to expected deforestation from 2000 to 2015 in *j* that are eligible for credits. The second term gives the total forest biomass CO_2_e in forest area expected to be lost in *j* from 2000 to 2015 due to cropland conversion. The credit limit *avoid_j_* could be higher if the credit limit formula included the emissions from forest expected to be cleared for urban use. We assume that an avoided emission credit would be in force up to 2020. However, because we assume that none of these emissions can be realistically avoided, *avoid_j_* is only equal to the cropland sector's share of 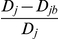. See column A in Table S21 for a list of countries where *D_j_*>*D_jb_* under each scenario and column B for *avoid_j_* values under each scenario.

To determine what portion of *avoid_j_* that *j* would sell we need information on 1) the net price of a biomass CO_2_e credit for the period 2000 to 2020 and 2) the opportunity cost of keeping forested parcels forested instead of cleared for agriculture. Let *p* – *c* be the net price of a biomass CO_2_e credit for the period 2000 to 2020 where *c* includes all program costs associated with a credit that are incurred by the landowner. In a research environment without data limitations we would determine two unique values for each cell forested as of 2000: 1) the net revenues associated with keeping the grid cell in forest up to 2020, including the net value of the avoided deforestation credit and 2) the net returns associated with clearing the grid cell at some point between 2000 and 2015 for crop production up to the year 2020. Then, assuming each grid cell manager is a net revenue maximizer, we would assign avoided emission credits to each grid cell where avoided deforestation behavior maximized the manager's net revenues (subject to the credit cap).

Due to data limitations, however, we only generate a country-level estimate of net returns to agriculture and therefore cannot make a parcel-by-parcel comparison of net returns to maintaining forest cover versus net returns to clearing and cropland use (although we could have used the cropland suitability layer in an attempt to diffuse average net returns across a country) Instead, we calculate the expected net present value (NPV) of all projected deforestation for cropland use in *j* according to a scenario by multiplying *A_j_* by *V_j_*, the average per hectare expected NPV of converting forested area in *j* to cropland use in 2010 for use up to 2020 ([7], [117], [118], Text S1, and column D of Table S21). We assume that *V_j_* is equal to 15% of the present value of agricultural revenues in country *j* [118]. If,
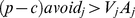
(17)or
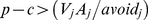
(18)then the country would, on average, earn more from forestalling all deforestation that produces *avoid_j_* until 2020. In other words, we assume that all cropland that emerges on forested land as of 2000 in *j* under a scenario is avoided if inequality (18) is met. See column G in Table S21 for the right hand side of inequality (18) for each eligible country *j* under each scenario. We model *p* – *c* values of $5 and $150 per Mg of CO_2_e.

## Supporting Information

Text S1 (0.37 MB DOC)

Figure S1 Biomass carbon content on land converted to urban or cropland use between 2000 and 2015.
(0.21 MB JPG)

Figure S2 Urban suitability map.
(0.24 MB JPG)

Figure S3 Areas where significant irrigation use is assumed if the grid cell is in cropland use.
(0.37 MB JPG)

Figure S4 Cropland suitability map for the *country* scenario. Scores have been normalized within a country so cross country comparisons are not appropriate.
(0.45 MB JPG)

Figure S5 Cropland suitability map for the *regional* scenario. Scores have been normalized within a region so cross region comparisons are not appropriate.
(0.49 MB JPG)

Figure S6 Net loss of biomass carbon between 2000 and 2015 due to LULC change. Results are summarized at the country-level and presented in Mg ha^−1^ units.
(0.36 MB JPG)

Table S1 Irrigated and rainfed cropland grid cell area in 2000 and 2015 under both scenarios.
(0.09 MB XLS)

Table S2 Urban grid cell area in 2000 and 2015 under both scenarios.
(0.07 MB XLS)

Table S3 Regions and their member countries in the *regional* scenario.
(0.03 MB XLS)

Table S4 Change in cropland grid cell area under the *country* scenario.
(0.07 MB XLS)

Table S5 Crop grid cell area growth rate for each region under the *regional* scenario.
(0.03 MB XLS)

Table S6 Change in cropland grid cell area under the *regional* scenario.
(0.04 MB XLS)

Table S7 Estimates of equation (10) in Text S1.
(0.04 MB XLS)

Table S8 Observed irrigation intensity in grid cells assigned irrigated cereal yield by country.
(0.06 MB XLS)

Table S9 The fraction of cropland grid cell area in each cropping zones under both scenarios.
(0.07 MB XLS)

Table S10 The average yield of each crop or crop group in 2000 by country (Mg ha^−1^).
(0.07 MB XLS)

Table S11 Expected growth in yields by crop or crop group and country.
(0.06 MB XLS)

Table S12 Calories per crop or crop type.
(0.05 MB XLS)

Table S13 The relative difference in cropland suitability in 2015 versus 2000 by country.
(0.06 MB XLS)

Table S14 The relative mix of crops or crop groups produced in each country in 2000: *q* = year 2000 crop mix.
(0.07 MB XLS)

Table S15 Alternative 2015 crop mix by country: *q* = 2 (“Low” 2015 harvested hectare mix).
(0.07 MB XLS)

Table S16 Alternative 2015 crop mix by country: *q* = 3 (“Mean” 2015 Harvested Hectare Mix).
(0.07 MB XLS)

Table S17 Alternative 2015 crop mix by country: *q* = 4 (“Mean” 2015 harvested hectare mix).
(0.07 MB XLS)

Table S18 Alternative 2015 crop mix by country: *q* = projected 2015 crop mix.
(0.07 MB XLS)

Table S19 Average water yield on rainfed cropland in 2000 and under both scenarios by country.
(0.05 MB XLS)

Table S20 Average biomass carbon storage by forest type by country in 2000 (Mg of carbon equivalent ha^−1^).
(0.05 MB XLS)

Table S21 Avoided emissions analysis.
(0.03 MB XLS)
