# Supplementary material for: Projecting Global Land-Use Change and Its Effect on Ecosystem Service Provision and Biodiversity with Simple Models
Source: PLoS One. 2010 Dec 15;5(12):e14327. doi: 10.1371/journal.pone.0014327 (PMC3002265; doi:10.1371/journal.pone.0014327)
Supplement: Text S1 — (0.37 MB DOC) [file pone.0014327.s001.doc]

**Text S1**

**The baseline land use / land cover (LULC) map**

The baseline global map of LULC in 2000 was constructed by integrating two raster datasets in a GIS environment using the following techniques.

1. A modified version of the Global Landcover 2000 (GLC2000) raster dataset. This dataset has a native spatial resolution of 1 km at the equator and identifies 22 LULC types. Three of these LULC types are cropland and mixed cropland land covers, and one includes artificial surfaces and associated areas (see <http://ies.jrc.ec.europa.eu/global-land-cover-2000> for details on modified GLC2000 LULC types). The modified version of GLC2000 came from Hoekstra et al. (2005).
2. The Global Urban-Rural Mapping Project (GRUMP), Alpha Version 2000 Urban Extents Dataset (http://sedac.ciesin.columbia.edu/gpw/). This dataset identifies urban land areas and has a native spatial resolution of 30 arc-seconds.
3. We resampled the integrated raster at a resolution of 5 km at the equator in order to match the spatial extent of the coarsest raster used in this analysis (the Gridded Population of the World, Version 3: Population Density; see below).

The LULC categories identified on the baseline global map include,

1. Urban: These areas were created by combining areas classified as artificial surfaces and associated areas in the modified GLC2000 map with areas indicated as urban in the GRUMP urban extents map.
2. Cropland: These areas were identified by combining the three cropland cover types identified in the modified GLC2000 map into one cropland category. Urban areas from the GRUMP map that coincided with modified GLC2000 cropland areas were reclassified as urban lands.
3. Other: All grid cells that are not in urban or cropland use or covered by water according to the modified GLC2000 map and the GRUMP urban extents map were assigned their modified GLC2000 LULC classes.
4. Protected areas: To identify which grid cells were in protected areas (IUCN categories I through VI) we used spatially-explicit vector data from the World Database on Protected Areas (http://www.unep-wcmc.org/wdpa/index.htm). Polygon data were available for most protected areas. However, some protected area locations were only available in point format. In such cases, we created polygons for these areas by assuming the protected range was circular and buffering these points with a radius that set the circle’s area equal to the areal extent of the protected area in the dataset’s attribute table. We identified grid cells as protected areas if they intersected any part of a protected are polygon.

The 2000 landcover map used in this analysis is available from the authors upon request.

**Calculating change in urban area from 2000 to 2015 country by country**

First, we collected 2000 urban population, *up00_j_*, and expected 2015 urban population, *up15_j_*, data for all countries *j* = 1, 2, …, *J* from UN (2008). Second, after calculating aggregate urban hectares in country *j* from the baseline 2000 LULC map, *ua00_j_*, we calculated *j*’s 2000 urban population density, *upd00_j_* = *up00_j_* / *ua00_j_* (people urban ha^-1^). Third, assuming urban population density does not change from 2000 to 2015, urban area in country *j* in 2015, *ua15_j_*, is given by the following,

$ua15_{j}=\left\{\begin{matrix} \begin{matrix} ua00_{j} \text{if} up15_{j}<up00_{j} \\ \end{matrix} \\ \frac{up15_{j}}{upd00_{j}} \text{if} up15_{j}\geq up00_{j} \end{matrix}\right.$. (S1)

Several countries, primarily former Soviet republics, have *up15_j_* < *up00_j_*. We assumed that these countries would not actually lose urban area and instead set *ua15_j_* = *ua00_j_* in these countries. Let the relative change in urban area in country *j* from 2000 to 2015 be given by *ua15_j_* / *ua00_j_*.

In Table S1 the values of *up00_j_*, *ua00_j_* (the variable *U_j_* in the text), *upd00_j_*, *up15_j_*, and *ua15_j_* for each *j* are given.

**Spatially allocating change in urban area country by country**

Our urban area suitability layer is a function of two maps. A slope suitability raster was generated in a GIS using a global 30 arc-second digital elevation model, GTOPO30, available from the USGS ([http://eros.usgs.gov/#/Find_Data/Products_and_Data_Available/gtopo30_info](http://eros.usgs.gov/#/Find_Data/Products_and_Data_Available/gtopo30_info)). Assuming that areas with higher slopes are less suitable for urban land uses, each raster cell was given a score ranging from one to five where higher values indicate lower slopes. Specifically, grid cells with slopes of less than 12% received values of 5, of between 12 and 18% received values of 4, of 18 to 30% received values of 3, of between 30 and 50% received values of 2, and of 50% or greater received values of 1. In order to force urbanization after 2000 into areas predicted to have high urban densities as of 2015, we also generated a raster based on predicted 2015 population densities. To do this, we reclassified a predicted population density raster for 2015 (Gridded Population of the World, Version 3: Population Density; <http://sedac.ciesin.columbia.edu/gpw/global.jsp>) on a one to five scale such that pixels with a population density of zero received values of 1 and values greater than one received values ranging from 2-5 using an equal interval system where areas with higher predicted densities were given scores closer to five.

To spatially allocate 2000 to 2015 growth in urban area we used an existing Idrisi Andes GIS land-change module known as GEOMOD (Hall et al. 1995, Pontius et al. 2001). GEOMOD is a grid-based simulation model that predicts changes in the spatial pattern of a particular LULC category across a time-step and region given an initial grid of the modeled LULC category in the region (e.g., urban area map for 2000), a suitability layer, and the amount of LULC area needed in the region at the end period (i.e., 2015). GEOMOD generally allocates LULC change according to the suitability layer, but, as a cellular automata model, is typically constrained by a neighborhood limitation. For example, a year 2000 map of urban areas in Canada, a grid indicating land suitability for urbanization, and an estimate of total Canadian urban area in 2015 are the three datasets needed by GEOMOD to predict the spatial distribution of Canadian urban growth from 2000 to 2015.

In the current study, we used the baseline LULC raster dataset described above to identify the locations of urban land use in 2000 (the starting time). A second map identified country boundaries to divide the landscape into country strata (<https://international.ipums.org/international/gis.shtml>). We defined suitability using the slope and predicted population density suitability layers in GEOMOD. Slope and predicted population density were given equal weight when defining suitability of a grid cell (e.g., if a grid cell had a suitability score of 3 for slope and a 2 for projected density then its final suitability score is 2.5, or, following integration in GEOMOD, 25 on the 0-100 scale used by that module). See Figure S1 for the urban suitability map on the 0 to 100 scale.

GEOMOD allocates grid cells to a LULC type. Thus, the 2015 grid cell target that the program used is given by,

*ugc15_j_* = (*ua15_j_* / *ua00_j_*) $\times$ *ugc00_j_* (S2)

where *ugc15_j_* is the number of urban grid cells in country *j* by 2015 and *ugc00_j_* is the number of urban grid cells in country *j* in 2000 according to the baseline map. If *ugc15_j_* was greater than the number of cells in a country then *ugc15_j_* was set equal to the number of cells available in that country excluding water and protected areas in the country. We ran the urban land transformation simulation in GEOMOD in one year time steps from 2000 to 2015 where new urban grid cells were added to each *j* until the 2015 grid cell target was met.

In Table S1 we also give data on *ugc00_j_* and *ugc15_j_* and the total grid cell area of *ugc15_j_* for each *j* (given by $U'_{j}$in the text).

**Calculating change in cropland area from 2000 to 2015 country by country**

Global cropland area in 2015 was predicted twice: once under a scenario we will call the *country* scenario and another time under a scenario we will call the *regional* scenario.

*Country scenario*

For each country in the world we calculated the average annual growth in cropland between 1980 and 1990, between 1990 and 2000, and between 2000 and 2005. Let these average annual growth values be given by *cg*8090*_j_*, *cg*9000*_j_*, and *cg*0005*_j_*,

$cg8090_{j}=\frac{c90_{j}-c80_{j}}{10\times c80_{j}}$ (S3)

$cg9000_{j}=\frac{c00_{j}-c90_{j}}{10\times c90_{j}}$ (S4)

$cg0005_{j}=\frac{c05_{j}-c00_{j}}{5\times c00_{j}}$ (S5)

where *c*XX*_j_* indicates the hectares in arable land and permanent crops in country *j* in the year XX. All data are from FAO (2008). According to the FAO (2008), “[a]rable land refers to land under temporary crops (double-cropped areas are counted only once), temporary meadows for mowing or pasture, land under market and kitchen gardens and land temporarily fallow (less than five years). The abandoned land resulting from shifting cultivation is not included.” Further, according to the FAO (2008), “[p]ermanent crops are sown or planted once, and then occupy the land for some years and need not be replanted after each annual harvest, such as cocoa, coffee and rubber. This category includes flowering shrubs, fruit trees, nut trees and vines, but excludes trees grown for wood or timber.”

Then for each country *j* we took the median value of the set of values formed by *cg8090_j_*, *cg9000_j_*, and *cg0005_j_* (if a country is missing one or two of these values the median is based on the values that exist). Let a country’s median average annual change in cropland hectares between 1980 and 2005 be given by *cg_j_*. Let the expected cropland area in 2015 in country *j* be given by *c15_j_* where,

$c15_{j}=c05_{j}\left(1+10cg_{j}\right)$ (S6)

Several countries had *cg_j_* values that we decided were not appropriate for calculating *c15_j_* and were changed accordingly (see the table below). Finally, we calculated the ratio *c15_j_* / *c00_j_* for each *j*. This ratio, given as$\delta$in the text, gives the relative increase in cropland area from 2000 to 2015 in country *j* under the *country* scenario.

| **Country** | *cg_j_* **value calculated** | *cg_j_* **value used** | **Notes** |
| --- | --- | --- | --- |
| China | 0.013 | 0.005 |  |
| Estonia | –0.059 | –0.025 |  |
| Lithuania | –0.067 | –0.028 |  |
| Croatia | –0.045 | –0.010 |  |
| Eritrea | 0.027 | 0.019 |  |
| Ethiopia | 0.060 | 0.035 |  |
| Western Sahara |  | 0 | No cropland on baseline map |
| Swaziland |  | 0 | No cropland on baseline map |
| Qatar |  | 0 | No cropland on baseline map |

In Table S3 the values of *c00_j_*, *c15_j_*, and *c15_j_* / *c00_j_* ($\delta_{j}$) for each country *j* are given.

*Regional scenario*

In this scenario we first calculated low, mean, and high estimates of cropland area in 2015 for each global region as defined in OECD-FAO (2008). Some regions are comprised on one nation (e.g., the U.S.); others are comprised of multiple countries (e.g., the European Union). See Table S4 for the roster of countries in each multi-country region.

The low estimate of 2015 cropland area in region *r*, *lowc15_r_*, is given by,

$lowc15_{r}=\left\{\begin{matrix} \begin{matrix} d15_{r}+\left[0.9\left(c00_{r}-d00_{r}\right)\right] \text{if} d15_{r}>d00_{r} \\ \end{matrix} \\ d15_{r}+\left[0.8\left(c00_{r}-d00_{r}\right)\left(d15_{r}/d00_{r}\right)\right] \text{if} d15_{r}\leq d00_{r} \end{matrix}\right.$ (S7)

where *d00_r_* is the total observed hectares in rice, wheat, coarse grains, and oil seeds in region *r* in the year 2000 according to OECD-FAO (2008), *d15_r_* is the total expected hectares in rice, wheat, coarse grains, and oil seeds in region *r* in the year 2015 according to OECD-FAO (2008), and *c00_r_* indicates all cropland hectares in region *r* in the year 2000 (FAO 2008) When calculating *lowc15_r_* we assume that regions that are expected to undergo an increase in total rice, wheat, coarse grain, and oil seed area between 2000 and 2015 (i.e., *d15_r_* > *d00_r_*) will experience a 10% decrease in all other cropland area between 2000 and 2015 (i.e., 0.9[*c00_r_* – *d00_r_*]). Otherwise, regions that are expected to undergo a decrease in total rice, wheat, coarse grain, and oil seed area between 2000 and 2015 (i.e., *d15_r_* $\leq$ *d00_r_*) will experience a ((1 – (0.8(*d15_r_* / *d00_r_*)))100)% decrease in all other cropland area between 2000 and 2015.

The mean estimate of 2015 cropland area in region *r*, *meanc15_r_*, is given by,

$meanc15_{r}=\left\{\begin{matrix} \begin{matrix} d15_{r}+\left(c00_{r}-d00_{r}\right) \text{if} d15_{r}>d00_{r} \\ \end{matrix} \\ d15_{r}+\left[0.9\left(c00_{r}-d00_{r}\right)\left(d15_{r}/d00_{r}\right)\right] \text{if} d15_{r}\leq d00_{r} \end{matrix}\right.$. (S8)

When calculating *meanc15_r_* we assume that regions that are expected to undergo an increase in total rice, wheat, coarse grain, and oil seed area between 2000 and 2015 (i.e., *d15_r_* > *d00_r_*) will experience no change in all other cropland area between 2000 and 2015 (i.e., *c00_r_* – *d00_r_*). Otherwise, regions that are expected to experience a decrease in total rice, wheat, coarse grain, and oil seed area between 2000 and 2015 (i.e., *d15_r_* < *d00_r_*) will experience a ((1 – (0.9(*d15_r_* / *d00_r_*)))100)% decrease in all other cropland area between 2000 and 2015.

The high estimate of 2015 cropland area in region *r*, *highc15_r_*, is given by,

$highc15_{r}=\left(d15_{r}/d00_{r}\right)c00_{r}$ (S9)

When calculating *highc15_r_* we assume that the rate of cropland area change across all crops is equal to the expected relative change in total rice, wheat, coarse grain, and oil seed area between 2000 and 2015.

Next we calculated the ratios *lowc15_r_* / *c00_r_*, *meanc15_r_* / *c00_r_*, and *highc15_r_* / *c00_r_* for each *r* and assigned each *r* its *lowc15_r_*, *meanc15_r_*, or *highc15_r_* ratio value (its$\delta_{r}$). Generally, we assigned regions comprised of developed nations its *lowc15_r_* ratio value and regions comprised of developing nations its *highc15_r_* ratio value. Let the chosen ratio for region *r* be given by $\delta_{r}$ = *c15_r_* / *c00_r_*.

In Table S5 the values of *d00_r_*, *c00_r_*, *d15_r_*, *lowc15_r_*, *meanc15_r_*, *highc15_r_* , and the chosen ratio, $\delta_{r}$= *c15_r_* / *c00_r_*, for each region *r* are given.

**Spatially allocating change in cropland area**

To spatially allocate new cropland (or abandoned cropland) we first generated a global cropland suitability grid map for each scenario. On this map each grid cell received a 1 to 5 score where higher numbers indicated greater crop productivity potential. Potential productivity was based on expected yields of multi-cropped cereals produced under intensive management.

The first step in generating the suitability layer required the estimation of a model that explains the density of irrigation use in each gird cell in a country in 2000. We explain the density of irrigation use in grid cell *x* in country *j* in 2000 with the following model,

$I_{xj}=\gamma_{0j}+\gamma_{1j}RFY_{xj}+\gamma_{2j}\left(IY_{xj}-RFY_{xj}\right)$ (S10)

where *I_xj_* $\in$[0,1] indicates the proportion of grid cell *x* in country *j* that was equipped for irrigation in 2000 (Aquastst 2007; <http://www.fao.org/nr/water/aquastat/irrigationmap/index10.stm>), *RFY_xj_* is the potential per hectare yield of multi-cropped rain-fed cereals managed under intensive input use in cell *x* in *j* (plate 53 in the GAEZ database; <http://www.iiasa.ac.at/Research/LUC/GAEZ/index.htm>), and *IY_xj_* is the potential per hectare yield of multi-cropping irrigated cereals managed under intensive input use in cell *x* in *j* (plate 54 in the GAEZ database). The$\gamma$ coefficients in equation (10) were estimated for each country *j* with ordinary least squares. Therefore, each country *j* has a unique set of estimated coefficients, given by$\hat{\gamma}_{0j},\hat{\gamma}_{1j}$, and$\hat{\gamma}_{2j}$.

The variable *I_xj_* will be low in a grid cell for one of two reasons: little to no agriculture existed in the cell in 2000 or its agriculture was primarily rainfed. We hypothesize that *I_xj_* tends to be lower in cells without much latent capacity for agricultural production. Therefore, all else equal we hypothesize that $\hat{\gamma}_{1j}$ < 0 for all *j*. Conversely, we hypothesize that as the yield bonus due to irrigation increases in *x* in country *j* (i.e., *IY_xj_* – *RFY_xj_* gets larger), farmers in cell *x* and *j*’s government are more likely to devote the resources necessary to equip a greater percentage of the area in *x* for irrigation, i.e., $\hat{\gamma}_{2j}$ > 0 for all *j*. We include all grid cells in a country when estimating equation (S10), not just those with cropland as of 2000. Therefore, the estimates of $\hat{\gamma}_{0j},\hat{\gamma}_{1j}$, and$\hat{\gamma}_{2j}$are biased downward compared to the estimates that would be generated if we only used grid cells with cropland as of 2000. See Table S6 for estimates of$\hat{\gamma}_{0j},\hat{\gamma}_{1j}$, and$\hat{\gamma}_{2j}$ for each *j*.

Then for every grid cell *x* in country *j* we used *RFY_xj_* and *IY_xj_* (data from plates 53 and 54) and the estimated equation (S10) to predict *I_xj_*. Let the prediction of *I_xj_* be represented by$\hat{I}_{xj}$. In general, cells with combinations of *RFY_xj_* and *IY_xj_* – *RFY_xj_* that were associated with higher *I_xj_* in *j* in 2000 (a significant amount of the area in the cell is irrigated) will be predicted to have higher$\hat{I}_{xj}$.

Within each country *j* we binned the vector of $\hat{I}_{xj}$values into 5 quantile sets (i.e., if *j* has 10 parcels then the parcels with the 2 lowest $\hat{I}_{xj}$ values are binned together, the 2 parcels with the 3^rd^ and 4^th^ lowest $\hat{I}_{xj}$ values are binned together, etc.). Then, we assigned *RFY_xj_* to parcel *x* if its $\hat{I}_{xj}$value was in one of the country’s three lowest bins of $\hat{I}_{xj}$ values and *IY_xj_* to all other *x*. Under the method used here the irrigation yield was assigned to 40% of all cells in a country. In a more detailed analysis we would have varied this assignment rule by country. For example, if irrigation is relatively rare in a county then we could have assigned irrigation yields only to cells with $\hat{I}_{xj}$in the highest bin. Alternatively, we could only given irrigated yields to those grid cells with a $\hat{I}_{xj}$ value that met some threshold, e.g., 60% or more of the grid cell area is predicted to be irrigated. See Table S7 for a comparison of area equipped for irrigation in grid cells assigned the irrigated yield versus those assigned the rainfed yield. The data in the table indicates that for almost all countries we assigned the irrigated yield to grid cells that, on average, had a much higher density of irrigation equipment as of 2000. See Figure S7 for a map of grid cells that were assigned their irrigated yields versus their rainfed yields.

Next, we reclassified the combination of slope and potential cereal yield in each cell on a scale of 1 to 5 to create a cropland suitability map where cells with higher potential yield and that were flatter received higher scores. Then suitability scores were normalized at the country level for the *country* scenario and at the regional level for the *regional* scenario to create two cropland suitability maps derived from the original suitability layer. GEOMOD internally converted grid cell scores from a 1 to 5 range to a 0 to 100 range on both cropland suitability maps. See Figures S5 and S6 for the *country* and *regional* scenario cropland suitability maps, respectively, on the 0 to 100 scale.

To spatially allocate new cropland in *j* or *r* under the country or regional scenario, respectively, we again used GEOMOD. Because GEOMOD allocates grid cells to a LULC type, the 2015 cropland target that the program used for the *country* scenario is given by,

*cgc15_j_* = (*c15_j_* / *c00_j_*) $\times$ *cgc00_j_* (S11)

where *cgc15_j_* is the target cropland grid cells in country *j* by 2015 and *cgc00_j_* is the number of cropland grid cells in country *j* in 2000 according to the baseline map. See Table S3 for *cgc00_j_* and *cgc15_j_* for each *j*

To complete the 2015 *country* scenario LULC map we added new cropland grid cells in arable land using GEOMOD in one year time steps in each country to the base LULC map that included with 2000 to 2015 urban expansion. The resulting 2015 raster met cropland grid cell targets under the *country* scenario in each country or, where a country’s available arable grid cells were insufficient to meet this target, included all available arable land in that country. Grid cells with higher scores in the *country* scenario cropland suitability raster were more likely to be selected as new cropland. Some arable grid cells were not available for conversion. These included arable grid cells in protected areas or urban use in 2000 or 2015 (the arable area in country *j* less 2000 or 2015 urban area and protected areas is given by$\bar{C}_{j}$in the text). We defined a grid cell as arable if at least one crop managed under irrigated conditions in the cell could be expected to achieve at least 40% of the crop’s global maximum irrigated yield using a subset of global maps of suitable cropland ranges for various crops and management regime combinations (Michael Jennings, *personal communication*). Cells that did not meet this criterion were not identified as arable and were excluded from the simulation.

In many countries, we needed to create more new cropland grid cells than indicated by the cropland grid cell difference indicated by *cgc15_j_*  – *cgc00_j_* to replace year 2000 cropland gird that were lost to urbanization between 2000 and 2015 in the urban expansion allocation step. See Table S3 for the aggregate grid cell area of *cgc00_j_* and *cgc15_j_* in each *j* (given by *C_j_* and $C'_{j}$in the text) and the fraction of *cgc15_j_* that was established after 2000 where *ncgc15_j_* indicates grid cell area established in crops after 2000 (given by $nC'_{j}$in the text) and *ocgc15_j_* indicates the amount established prior to 2000 that remains as of 2015 following the urban simulation (given by $oC'_{j}$in the text).

The grid cell target used for the *regional* scenario is given by,

*cgc15_r_* = (*c15_r_* / *c00_r_*) $\times$ *cgc00_r_* (S12)

where *cgc15_r_* is the target cropland grid cells in region *r* by 2015 and *cgc00_r_* is the number of cropland grid cells in region *r* in 2000 according to the baseline map (the area of *cgc00_r_* in region r is given by *C_r_* in the text). To complete the 2015 *regional* scenario LULC map we used GEOMOD to add new cropland grid cells in arable land in one year time steps in each region to the base LULC raster that included 2000 to 2015 urban expansion. Grid cells with higher scores on the *regional* scenario cropland suitability map were more likely to be selected for new cropland. In the final 2015 cropland raster, grid cell targets were met in each region or, where sufficient land was unavailable, all available arable grid cells were converted (the final area of cropland grid cells in region *r* in 2015 is given by$C'_{r}$in the text). Again, protected area and urban area grid cells were not available for conversion (the arable area in country *j* less 2000 or 2015 urban area and protected areas is given by$\bar{C}_{r}$in the text).

See Table S8 for the total cropland grid cell area in each country *j* after *cgc15_r_* has been spatially allocated. Because some grid cells in cropland in 2000 could have been lost to urban uses by 2015 the number of cropland grid cells on the 2015 *regional* scenario map established after 2000 could be larger than cropland grid cell difference indicated by *cgc15_r_* – *cgc00_r_*. Also see Table S8 for the fraction of cropland that was established after 2000 by country under the *regional* scenario where *ncgc15_j_* indicates grid cell area established in crops after 2000 (given by $nC'_{j}$in the text) and *ocgc15_j_* indicates the amount established prior to 2000 that remains as of 2015 (given by $oC'_{j}$ in the text).

Finally, where the area of agriculture is predicted to decline in a country or region (i.e., *c15_j_* / *c00_j_* < 1 or *c15_r_* / *c00_r_* < 1), the county or region’s 2015 cropland grid cell target is met by removing some grid cells from agriculture. In many cases, some or all of the required grid cell loss was achieved through cropland grid cell conversion to urban use in the urban growth allocation process. Any targeted cropland grid cell loss that was not accounted for in urban growth allocation stage was met by allocating abandoned cropland on a scenario map. In these cases grid cells with the lowest cropland suitability scores were most likely to be removed from agriculture.

**Calculating cropping capability shares by country**

We used the map “Multiple cropping zones - rain-fed conditions” (plate 13) from the Global Agro-ecological Zones (GAEZ) mapping project (http://www.iiasa.ac.at/Research/LUC/GAEZ/index.htm) to determine the fraction of each country’s cropland grid cell area that was in single, double, or triple-cropping zones. “Limited double cropping”, “undefined”, and “no cropping” areas on the map were assumed to be single cropping areas; otherwise all cropping categories were indicated by their name. We calculated these fractions by country for the 2000 baseline map (given by$\gamma_{j}$, $\lambda_{j}$, and 1–$\gamma_{j}$–$\lambda_{j}$in the text), the *country* scenario 2015 LULC map (for all, new, and old cropland; given by$\gamma'_{j}$, $\lambda'_{j}$, and 1–$\gamma'_{j}$–$\lambda'_{j}$; $n\gamma'_{j}$, $n\lambda'_{j}$, and 1–$n\gamma'_{j}$–$n\lambda'_{j}$; and $o\gamma'_{j}$, $o\lambda'_{j}$, and 1–$o\gamma'_{j}$–$o\lambda'_{j}$in the text), and the *regional* scenario 2015 LULC map (for all, new, and old cropland; given by$\gamma'_{j}$, $\lambda'_{j}$, and 1–$\gamma'_{j}$–$\lambda'_{j}$; $n\gamma'_{j}$, $n\lambda'_{j}$, and 1–$n\gamma'_{j}$–$n\lambda'_{j}$; and $o\gamma'_{j}$, $o\lambda'_{j}$, and 1–$o\gamma'_{j}$–$o\lambda'_{j}$in the text). See Table S9 for all country-level data.

**Calculating country-level cropland suitability scores**

For each country on the 2000 baseline map, the *country* scenario 2015 LULC map, and the *regional* scenario 2015 LULC map we calculated the average cropland suitability score of gird cells in new, old, and all cropland use. Recall that each scenario has a differently normalized cropland suitability layer. So we scored the baseline 2000 map with each suitability layer to make relevant 2000 to 2015 comparisons under each scenario. As noted above, GEOMOD produces suitability rasters from inputs that indicate grid cell suitability on a 0 to 100 scale. Thus, a country’s average agricultural suitability score ranges from 0 to 100 where a higher score indicates greater productivity. If a country’s average suitability score increases from 2000 to 2015 that means that, on average, the 2015 allocation of cropland in the country has shifted to more productive land as measured by potential (irrigated or rainfed) grain yield. Similarly, a country that experiences a reduction in mean suitability from 2000 to 2015 has shifted to use less productive land. In the text $n\beta_{j}$is the ratio of the average suitability on *j*’s new cropland to the average suitability of *j*’s cropland in 2000 and $o\beta_{j}$ is the ratio of the average suitability on *j*’s old cropland to the average suitability on *j*’s cropland in 2000. See Table S13 for $n\beta_{j}$ and $o\beta_{j}$ for each *j* under each scenario.

**Crop mix in 2015**

We define 15 crop type categories in this analysis and index them with *i* = 1, 2, …,13. See Table S11 for the individual crops that make up each crop category and calorie contents per 100 grams of each crop and the average calorie content across a crop category.

The number of hectares used to produce each crop category *i* in each country *j* in 2000 is from FAO (2008). Let *h00_ji_* indicate the number of hectares used for crop category *i* in country *j* in 2000. Therefore, the fraction of harvested hectares in *j* used for crop in 2000 is given by,

$\gamma00_{ji}=\left(h00_{ji}/\sum_{g=1}^{13} h00_{jg}\right)$ (S13)

where$\sum_{i=1}^{13} \gamma00_{ji}=1$. One of the simplest assumptions we can make is that the fraction of harvested hectares used for crop category *i* in 2015 in country *j*, given by$\gamma15_{ji}$, is equal to$\gamma00_{ji}$. In the text we set $\sigma_{ji}$= $\omega_{ji}$=$\gamma00_{ji}$ in the “Year 2000 crop mix” analysis. See Table S14 for $\gamma00_{ji}$for each crop type and country combination. Otherwise we can use data from OECD-FAO (2008) to set$\gamma15_{ji}$.

In OECD-FAO (2008) expectations for cropland area in 2015 are given for crop categories rice, wheat, coarse grains, and oil seeds. For all countries *j* that belong to region *r* let their harvested hectares in these crop types, indexed by *a* = 1, …,4, expand by,

$\hat{h}15_{ja}=\frac{d15_{ra}}{d00_{ra}}h00_{ja}$ (S14)

For the other crop types, indexed by *b* = 1, …., 9, we calculate low, mean, and high 2015 harvested hectare values. Let the low values of 2015 harvested hectares for crop type *b* be given by,

$low\hat{h}15_{jb}=\left\{\begin{matrix} \begin{matrix} 0.9\times h00_{jb} \text{if} d15_{r}>d00_{r} \\ \end{matrix} \\ 0.8\times h00_{jb}\times\frac{d15_{r}}{d00_{r}} \text{if} d15_{r}\leq d00_{r} \end{matrix}\right.$ (S15)

Let the mean values of 2015 harvested hectares for crop type *b* be given by,

$mean\hat{h}15_{jb}=\left\{\begin{matrix} \begin{matrix} h00_{jb} \text{if} d15_{r}>d00_{r} \\ \end{matrix} \\ 0.9\times h00_{jb}\times\frac{d15_{r}}{d00_{r}} \text{if} d15_{r}\leq d00_{r} \end{matrix}\right.$ (S16)

Let the high values of 2015 harvested hectares for crop type *b* be given by,

$high\hat{h}_{jb}=\frac{d15_{r}}{d00_{r}}\times h00_{jb}$ (S17)

Finally, the relative mix of harvested hectares in each crop category in 2015 using data from OECD-FAO (2008) for crop categories indexed by *a* are,

$low\gamma15_{ja}=\left(\hat{h}15_{ja}/\sum_{d=1}^{4} \hat{h}15_{jd}+\sum_{b=1}^{9} low\hat{h}15_{jb}\right)$ (S18)

$mean\gamma15_{ja}=\left(\hat{h}15_{ja}/\sum_{d=1}^{4} \hat{h}15_{jd}+\sum_{b=1}^{9} mean\hat{h}15_{jb}\right)$ (S19)

$high\gamma15_{ja}=\left(\hat{h}15_{ja}/\sum_{d=1}^{4} \hat{h}15_{jd}+\sum_{b=1}^{9} high\hat{h}15_{jb}\right)$ (S20)

and for crop categories indexed by *b* are ,

$low\gamma15_{jb}=\left(low\hat{h}15_{jb}/\sum_{a=1}^{4} \hat{h}15_{ja}+\sum_{e=1}^{9} low\hat{h}15_{je}\right)$ (S21)

$mean\gamma15_{jb}=\left(mean\hat{h}15_{jb}/\sum_{a=1}^{4} \hat{h}15_{ja}+\sum_{e=1}^{9} mean\hat{h}15_{je}\right)$ (S22)

$high\gamma15_{jb}=\left(mean\hat{h}15_{jb}/\sum_{a=1}^{4} \hat{h}15_{ja}+\sum_{e=1}^{9} high\hat{h}15_{je}\right)$ (S23)

for crop types indexed by *b*. Note that$\sum_{a=1}^{4} low\gamma15_{ja}+\sum_{b=1}^{9} low\gamma15_{jb}=1$, $\sum_{a=1}^{4} mean\gamma15_{ja}+\sum_{b=1}^{9} mean\gamma15_{jb}=1$, and $\sum_{a=1}^{4} high\gamma15_{ja}+\sum_{b=1}^{9} high\gamma15_{jb}=1$. See Tables S15-S17 for the $low\gamma15$, $mean\gamma15$, and $high\gamma15$fractions for each crop type and country combination.

We use one more crop mix distribution. Here we tweaked country-level mixes until global area in the four crop categories modeled in OECD-FAO (2008) were approximately equal to OECD-FAO (2008)’s predicted harvested hectare count for these our crops given the *regional* scenario’s final cropland area for each country *j*. This matrix of harvested hectare type weights is signified by$pmatch\gamma15$. In the text we set $\sigma_{ji}$= $\omega_{ji}$=$pmatch\gamma15_{ji}$in the “OECD-FAO (2008) mix” analysis. See Table S18 for $pmatch\gamma15$data.

**Calculating expected growth in yields from 2000 to 2015 for each crop type and country combination**

We used data from Rosegrant et al. (2001) and OECD-FAO (2008) to determine expected 2000 to 2015 growth in per hectare yield for each country and crop category combination. Expected yield in 2015 is given by $\alpha_{ji}Y_{ji}$where $\alpha_{ji}$is the 2000 to 2015 yield inflator of crop category *i* in country *j* due to technology growth and *Y_ji_* is observed 2000 yield. See Table S11 for data on$\alpha_{ji}$for each crop type and country combination. See Table S10 for data on *Y_ji_* for each crop category and country combination where yield is measured in Mg ha^-1^.

**The avoided emissions analysis**

Given concerns about additionality (only counting emissions that we had not expected to avoid in the future), countries will only be certified to sell avoided emission offsets if they are able to reduce deforestation below a baseline level. Therefore, in order to identify which nations would have been able to generate offsets beginning in 2000 we compare predicted national deforestation rates between 2000 and 2015 under the *country* and *regional* scenarios with a pre-2000 deforestation rate (the baseline); namely, the annualized rate of deforestation from 1990 to 2000 (FAO 2009). We consider a nation eligible to supply avoided emissions offsets if its predicted annual deforestation rate under a scenario, *D_j_*, is greater than the annualized deforestation rate from 1990 to 2000.

$D_{j}=\frac{F_{j}-F'_{j}}{15F_{j}}$ (S24)

where *F_j_* indicates the forested hectares in country *j* on the year 2000 baseline map and $F'_{j}$ indicates the forested hectares on a 2015 LULC scenario map (forested area is given by LULC categories 1 through 10 on the baseline and 2015 LULC scenario maps). Table S22 indicates the countries that would be eligible to sell REDD offsets under our two LULC change scenarios.

Having identified the nations with predicted 2000 to 2015 deforestation rates greater than observed 1990 to 2000 deforestation rates, we next quantify the expected amount of CO_2_e emissions from deforestation under each scenario for each eligible country. The amount of biomass carbon in the forests that are expected to be cleared between 2000 and 2015 under a scenario in country *j* is given by,

$totalC_{j}=\sum_{k=1}^{10} \left(F_{jk}-F'_{jk}\right)C_{jk}$ (S25)

where *k* = 1, …, 10 indexes the 10 forest types on the global LULC maps and *C_jk_* represents the average Mg of CO_2_e stored ha^-1^ of forest type *k* in country *j*. See Table S21 for the forest types and the carbon equivalent of *C_jk_* for each *j* and *k* combination. However, only a portion of this lost biomass carbon can be avoided in exchange for offsets; namely, the portion of the biomass carbon losses that are above and beyond the baseline rate of loss. Therefore, the maximum expected carbon losses that can be avoided in exchange for credits in country *j* is given by,

$eligibleC_{j}=\left(\left(D_{j}-D_{jb}\right)/D_{j}\right)totalC_{j}$ (S26)

So far we have not distinguished between the sources of forest conversion. We assume the opportunity cost of avoiding expected deforestation on grid cells expected to convert to urban use under a scenario will be much greater than any avoided emission offset payment. Therefore, we assume that none of the forest conversion due to urban expansion from 2000 to 2015 would have been avoided. This means that we only consider the portion of *totalC_j_* that is due to expected deforestation for cropland use (*avoid_j_* in equation (16) of the text).

$avoid_{j}=\left(\left(D_{j}-D_{jb}\right)/D_{j}\right)\sum_{k=1}^{10} A_{jk}C_{jk}$ (S27)

where *A_jk_* are the hectares of forest type *k* in country *j* in 2000 that are projected to be in cropland by 2015.

In some cases the value of avoided emissions on forested land being considered for conversion to cropland may be greater than the net value of crop production. In such cases we would expect the landowner to have chosen to forego expected deforestation for cropland in the period 2000 to 2015 in exchange for a payment. Assume grid cell *x* in country *j* has forested landcover *k* in 2000. We assume that conversion to crop production would occur in grid cell *x* in 2010 and that once converted, will be in production for 10 years. Let the expected net present value of crop *c*’s production from 2010 to 2020 in *x* (revenues less costs including revenues and costs generated by forest clearing) in country *j* be given by *V_jcx_* where *V_jx_* is equal to the highest *V_jcx_* value across all crops *c* = 1, 2, …, *C*. The owner of grid cell *x* in country *j* will avoid deforestation from 2000 to 2015 if,

$pG_{jx}A_{jx}\left(\sum_{k=1}^{10} I_{jxk}C_{jk}\right)+\psi_{x}+\xi_{x}-c_{x}>V_{jx}$ (S28)

where *p* is the price of a Mg of CO_2_e offset from 2000 to 2015, *G_jx_* equals 1 if grid cell *x* in country *j* is “scheduled” to convert from forest cover to cropland under a scenario and equals 0 otherwise, *A_jx_* is the hectares in grid cell *x*, *I_jxk_* equals 1 if grid cell *x* in country *j* is in forest type *k* in 2000 and equals 0 otherwise, $\psi_{x}$ is the option value of not clearing the forest, $\xi_{x}$ is the expected NPV of any revenue generated by the forest in grid cell *x* from 2000 to 2015 (this is revenue that has to be compatible with avoided emission program rules), and *c_x_* is the landowner’s costs for avoided emission program certification, property-right enforcement, and any other program fees. We expect all parcels in country *j* that meet inequality (S28) would accept an avoided emission offset unless the country’s *avoid_j_* cap is met first.

Unfortunately, we are missing much of the data need to calculate inequality (S28) for each grid cell in countries where *D_j_ > D_jb_*. First, we do not have data on $\psi_{x}$, $\xi_{x}$, or *c_x_*. Second, we cannot generate a specific *V_jx_* for each grid cell *x* because we do not have data on the grid cell-specific expected yields and production costs for each crop type and the expected net value of selling felled wood and other non-timber forest products generated during the clearing of grid cell *x*. Instead, using country-level data, we construct one *V* value for each country that does not consider the value of felled forest products. The *V* value for each country is assigned to all parcels in country *j*.

To estimate a country’s *V* value we first estimate average cropland revenues in the country. Let *R_j_* represent the present value of revenue generated by a grid cell in country *j* from 2010 to 2020,

$R_{j}=A_{j}\sum_{t=2010}^{2020} \frac{\left[\left(\sum_{c=1}^{C} p_{cjt}Y_{jc}h00_{jc}\right)/c00_{j}\right]}{1.07^{t-2000}}$ (S28)

where *A_j_* is the size of grid cells in hectares, *p_cjt_* is the price of crop *c* in country *j* in year *t*, *Y_jc_* is the observed year 2000 average per hectare yield of crop *c* in country *j*, *h00_jc_* is the number of hectares that produced *c* in *j* in year 2000, *c00_j_* is the cropland area in country *j* in the year 2000, and the discount rate is 7%. The term $\left[\left(\sum_{c=1}^{C} p_{cjt}Y_{jc}h00_{jc}\right)/c00_{j}\right]$ represents the average annual per hectare revenue from crop production in country *j* assuming year 2000 yields and crop production mixes. Note that we assume that agricultural yields will remain constant at year 2000 levels until 2020. In the analysis we assume per annum prices will grow according to the average per annum growth rate predicted for 2010 to 2015 in OECD-FAO (2009). We also use OECD-FAO (2009) to set 2010 prices for each *c* unless prices for type *c* are not given. For those products lacking price information (vegetables and melons, pulses, roots and tubers, fruit excluding melons, fiber crops, treenuts, and other cereals), we assume the revenue from production is equal to the minimum revenue generated by the production of products with known prices.

Finally, we generate a net present value for cropland use in parcel *x* of country *j* by assuming a profit margin of 15% (Stern 2007). In other words, *V_j_* = 0.15*R_j_* to transform the gross value of agriculture into the net value of agriculture.

**Validation**

We can compare our projections to the changes that have been observed so far beginning in the year 2000.

Let us consider urban area. In our projection urban **grid cell area** increases 50,600 square kilometers per annum from 2000 to 2015. First, recall that urban grid cell area is not the same as urban area; expansion in urban grid cell area does not equal expansion in urban area specifically as many grid cells that are primarily urban will include some other land covers as well. So the expansion in actual urban area in our projection is less than 50,600 square kilometers per annum.

One source estimates that from 2000 to 2005 global urban area expanded at a rate of 20,000 km^2^ per year (Holmgren 2006). However, this estimate only includes **some** urban expansion:

[t]he study is limited to urban areas with more than 100,000 inhabitants and does not include infrastructure developments. Presumably, smaller cities take up considerable areas, but perhaps it is the larger cities that represent the bulk of overall growth of urban areas. Obviously, infrastructure expansion is considerable, but no documentation of this could be obtained. Lacking further documented information on the topic, this study uses the figures directly from Angel et al. (2005).

Therefore, once we include the smaller cities of the world, all urban-related infrastructure, acknowledge that urbanization rates are increasing every year, and subtract a bit from our 50,600 square kilometers per annum rate to account for partially urbanized grid cells than our rate and the estimate from the study above began to converge.

We also downloaded Globcover_V2.2 from the European Space Agency (ESA; <http://ionia1.esrin.esa.int/>). Globcover_V2.2 gives global land cover as of 2005. However, there are some issues with comparing this map to GLC2000 (the source of our base year 2000 map). First, while some of the LULC types on Globcover_V2.2 are similar to those on GLC2000 there are some differences in LULC classification and no guidance on reconciling these differences. Second, even for LULC types that are the same across the maps, there are some obvious mapping incongruities. For example, both maps have a LULC category called “Artificial surfaces and associated areas.” This is the LULC that is supposed to include most urban areas and associated infrastructure. According to GLC2000, there was 273,885 square kilometers of artificial surface in 2000. According to Globcover_V2.2 there was 310,942 square kilometers of artificial surface in 2005. Part of the problem with this calculation is borne out by a country-by-country comparison between the GLC2000 and Globcover_V2.2. According to the two maps 78 countries **lose** artificial surfaces and associated area between 2000 and 2005; this is unrealistic as artificial areas are rarely if ever converted back to vegetation (the aggregate area lost across these 78 countries is -67,336 square kilometers).

Some of the expected urban growth may be accounted for in the LULC category “Bare Areas.” Both maps have this LULC category. Globally, this category saw a 1,061,390 square kilometer gain from 2000 to 2005 across the two maps, or 212,278 km^2^ per annum.

In general, given the dramatic differences in areas in the GLC2000 to Globcover_V2.2 comparison (probably due to very different mapping methodologies) more research is necessary to assess their role in validation. In this vein, the field has some way to go according to Potere et al. (2009), who compare eight different land use products and find great variation in their definition of urban area.

To validate our harvested hectares expansion predictions we can use UN FAO data. From 2000 to 2007 the area in “Arable land and Permanent crops” (the UN FAO’s category for working cropland) increased by 233,331 km^2^. If the 2000 to 2007 observed rate in arable land and permanent crop area continues as is there will be in increase of 500,000 km^2^ by 2015. In the *country* scenario we expect global harvested hectares to increase by 680,000 km^2^ and in the *regional* scenario we expect global harvested hectares to increase by 530,000 km^2^. Thus from a global perspective, the *regional* scenario is more consistent with FAO data for the 2000 to 2007 period. Interestingly, from 2000 to 2005 the area in arable land and permanent crops increased by 223,543 km^2^. Using this rate of harvested hectare expansion there would be 670,600 km^2^ of additional harvested hectares by 2015, much closer to the aggregate *country* scenario prediction. It appears the economic slowdown greatly affected the rate of cropland conversion from 2005 to 2007.

We also project that much of the gain in harvested hectares will come from countries with low HDI scores. UNFAO data bears this out as well. From 2000 to 2007 harvested hectares in “Least Developed Countries” and “Land Locked developing countries” grew by 10.8% and 10.3%, respectively. The global rate of harvested hectare expansion over this time period was 1.5%.

We can also compare our results to the Millennium Ecosystem Assessment’s (MA’s) scenarios analysis. The MA provides global maps of landcover for the years 2050 and 2100 (the digital maps can be downloaded at http://wdc.nbii.gov/ma/datapage.htm). Given the significant temporal gap between 2015 and 2050 a comparison of global LULC maps is inappropriate; even if our projections as of 2015 are significantly different than the MA’s projected 2050 map that does not mean the two projects disagree, the remaining 35 years could lead to convergence. Instead we can use graphs of global LULC area trajectory over time from chapter 9 of the Scenarios book of the MA to compare validate our results. For example, according to the MA, in 2000 global forest cover was 44.5 to 45.5 million km^2^ and 2015 global forest cover is projected to change anywhere from a loss of 2.25 million km^2^ to a 0.25 million km^2^ gain, depending on the scenario. In our analysis, global forest cover was 42.2 million km^2^ in 2000 and ranges from 38.6 to 39.4 million km^2^ in 2015. Therefore, we project a 2.8 to 3.6 million km^2^ loss in global forest cover from 2000 to 2015, slightly higher than the MA scenarios predictions.

Further, according to the MA, in 2015 global agricultural cover will range from 50.2 to 55.8 million km^2^, depending on the scenario; this projection includes crop and pasture lands. This represents a change of anywhere from -0.45 to 4.1 million km^2^. We project a 1.48 to 1.88 million km^2^ gain in cropland grid cell area. We do not model the expansion of pasture in our model. According to Steinfeld et al. (2006) pasture area expanded 0.2% per year globally from 1991 to 2000. If we extrapolate this rate out to 2015 we have a 1.4 million km^2^ of expansion in pasture grid cell area. If we add this to our 1.48 to 1.88 million km^2^ gain in cropland grid cell area we have a total expansion in agricultural grid cell area of 2.88 to 3.28 million km^2^**.** However, recall that not all agricultural grid cell area is actually in agricultural use so the true gain in agricultural area in our projection will fall below this range.

Finally, global irrigated area is projected to increase by about 0.5% to 6% from 2000 to 2015 according to the MA. In our analysis we project a 5.5 to 8.1% in grid cell area that will benefit to some degree by irrigation by 2015. Again since not all crops in a grid cell with irrigation capacity will benefit from irrigation the actual expansion of irrigation would be below the expansion of grid cell area.

To summarize, our projections of global LULC change as of 2015 are within the range of projections from the MA scenario analysis, the most elaborate and comprehensive global scenario analysis produced to date.

**References**

Angel S, Sheppard S, Civco D (2005). The Dynamics of Global Urban Expansion. The Word Bank, Transport and Urban Developemnt Department.

Holmgren P (2006) Global Land Use Area Change Matrix Input to the Fourth Global Environmental Outlook (GEO – 4). Forest Resources Assessment Working Paper - 134. UN FAO, Rome.

Lambin EF, Geist, HJ (2006) Land-Use and Land-Cover Change: Local Processes and Global Impacts (The IGBP Series) Berlin: Springer-Verlag, 222 p.

Potere D, Schneider A, Angel S, Civco D (2009). Mapping urban areas on a global scale: which of the eight maps now available is more accurate? International Journal of Remote Sensing 30, 6531-6558
