# Supplementary figures and images for: Projecting Global Land-Use Change and Its Effect on Ecosystem Service Provision and Biodiversity with Simple Models
Source: PLoS One. 2010 Dec 15;5(12):e14327. doi: 10.1371/journal.pone.0014327 (PMC3002265; doi:10.1371/journal.pone.0014327)

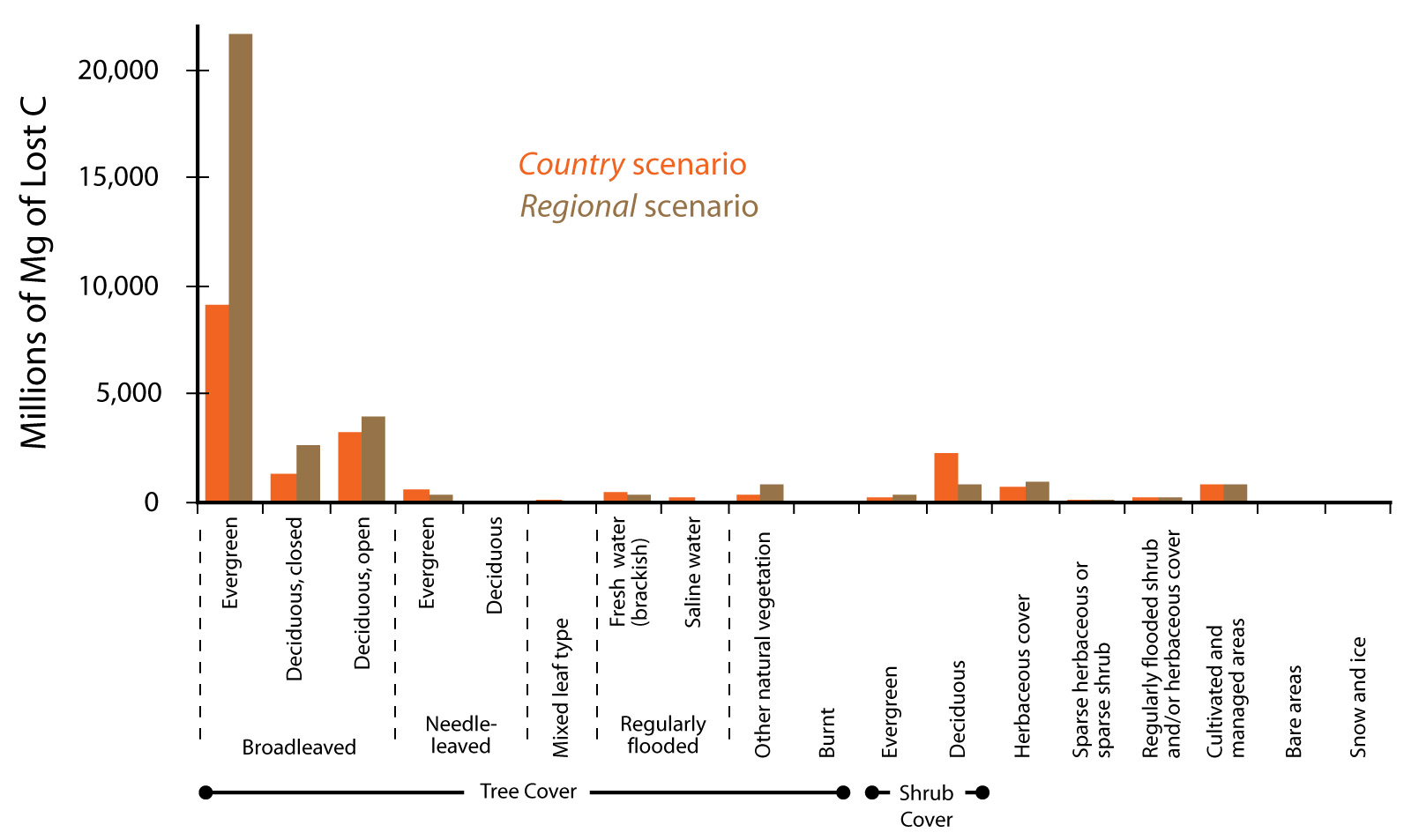

Supplement: Figure S1 — Biomass carbon content on land converted to urban or cropland use between 2000 and 2015. (0.21 MB JPG) [file pone.0014327.s002.jpg]

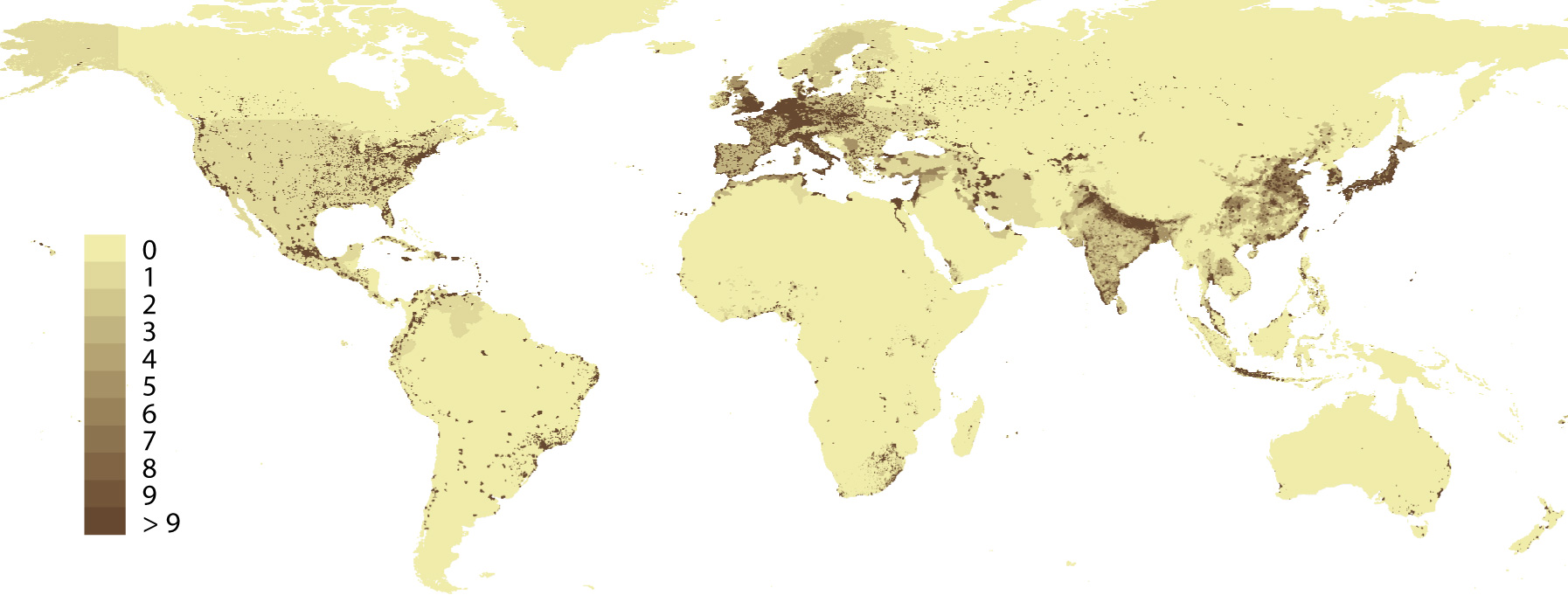

Supplement: Figure S2 — Urban suitability map. (0.24 MB JPG) [file pone.0014327.s003.jpg]

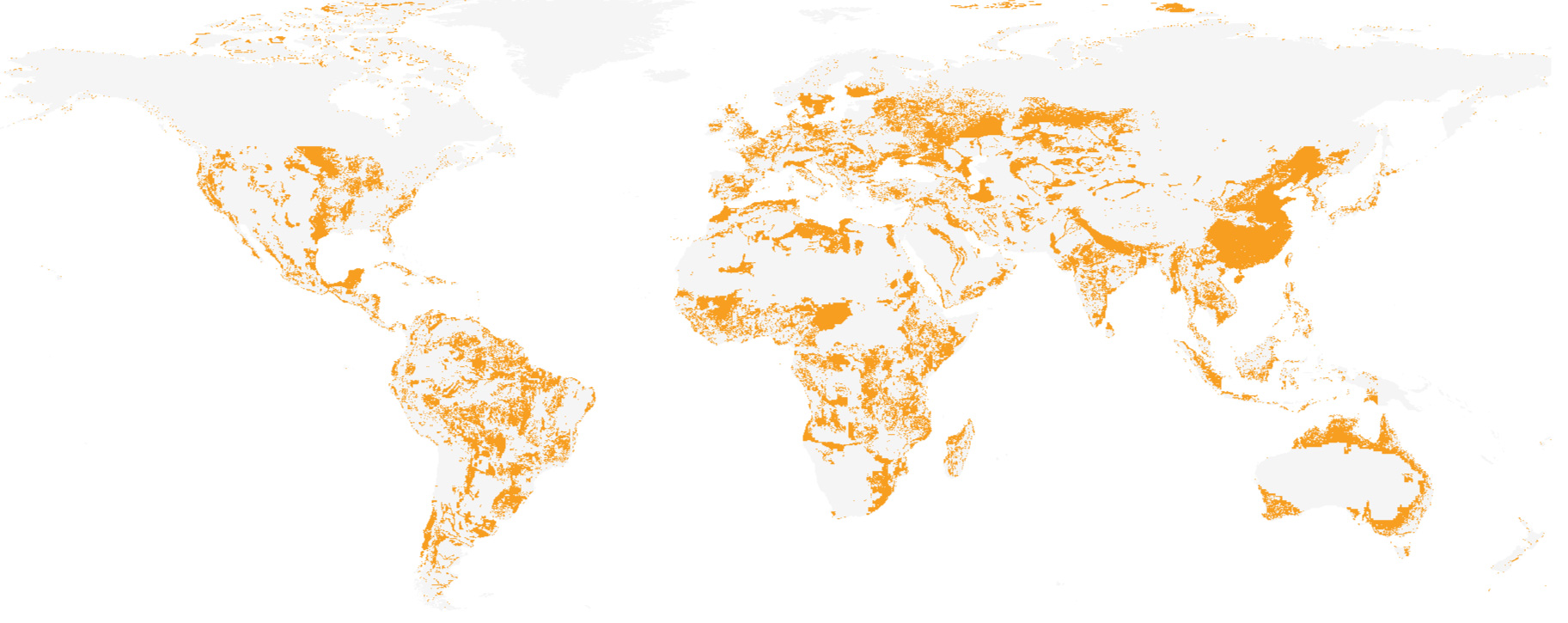

Supplement: Figure S3 — Areas where significant irrigation use is assumed if the grid cell is in cropland use. (0.37 MB JPG) [file pone.0014327.s004.jpg]

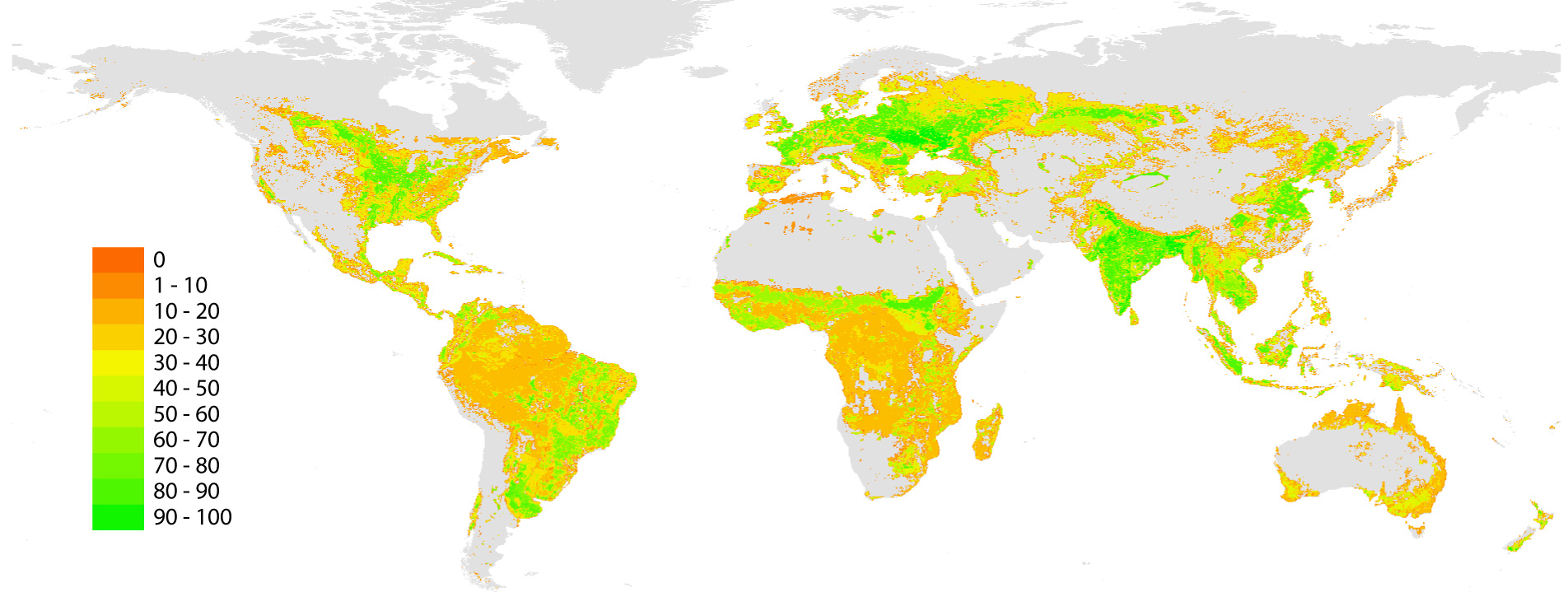

Supplement: Figure S4 — Cropland suitability map for the country scenario. Scores have been normalized within a country so cross country comparisons are not appropriate. (0.45 MB JPG) [file pone.0014327.s005.jpg]

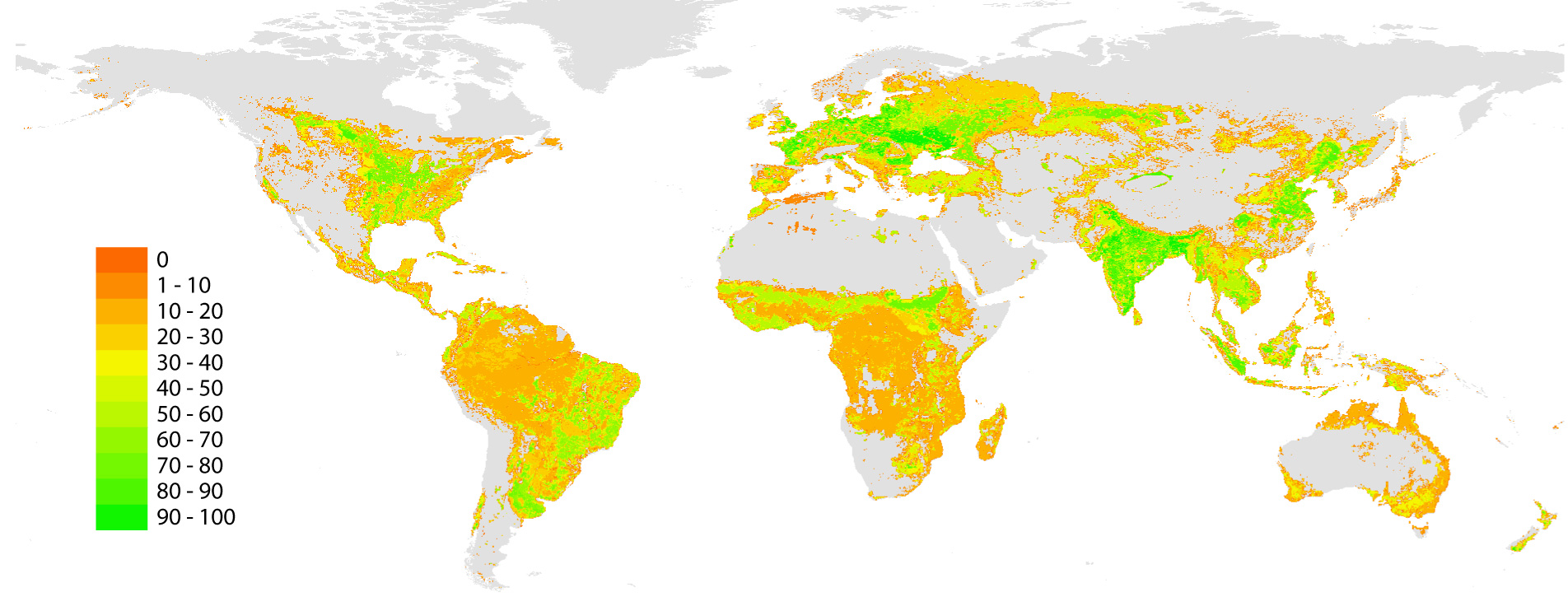

Supplement: Figure S5 — Cropland suitability map for the regional scenario. Scores have been normalized within a region so cross region comparisons are not appropriate. (0.49 MB JPG) [file pone.0014327.s006.jpg]

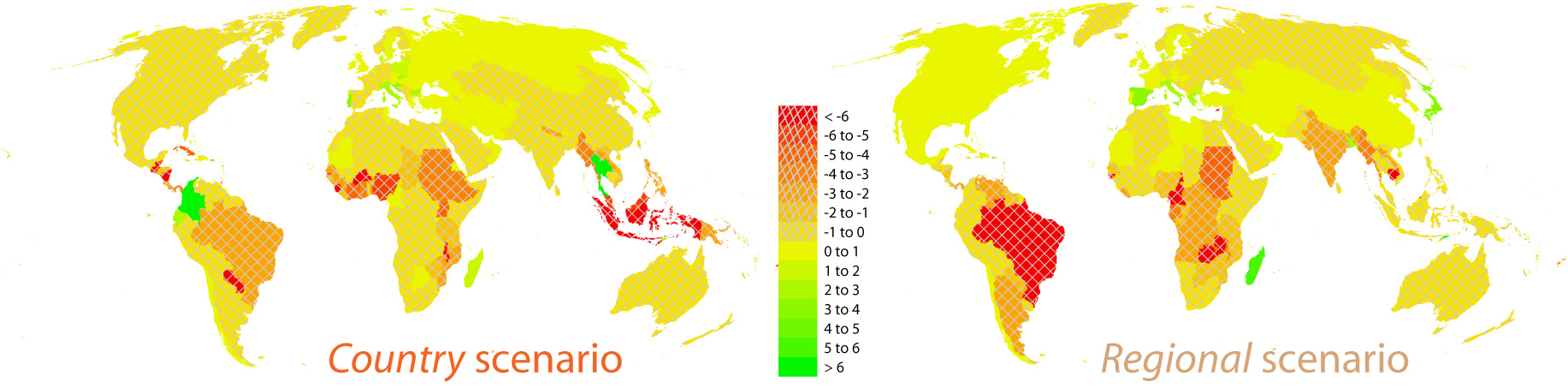

Supplement: Figure S6 — Net loss of biomass carbon between 2000 and 2015 due to LULC change. Results are summarized at the country-level and presented in Mg ha−1 units. (0.36 MB JPG) [file pone.0014327.s007.jpg]
